# Leisure Time Physical Activity of Moderate to Vigorous Intensity and Mortality: A Large Pooled Cohort Analysis

**DOI:** 10.1371/journal.pmed.1001335

**Published:** 2012-11-06

**Authors:** Steven C. Moore, Alpa V. Patel, Charles E. Matthews, Amy Berrington de Gonzalez, Yikyung Park, Hormuzd A. Katki, Martha S. Linet, Elisabete Weiderpass, Kala Visvanathan, Kathy J. Helzlsouer, Michael Thun, Susan M. Gapstur, Patricia Hartge, I-Min Lee

**Affiliations:** 1 Division of Cancer Epidemiology and Genetics, National Cancer Institute, Bethesda, Maryland, United States of America; 2 Epidemiology Research Program, American Cancer Society, Atlanta, Georgia, United States of America; 3 Department of Medical Epidemiology and Biostatistics, Karolinska Institute, Stockholm, Sweden; 4 Cancer Registry of Norway, Oslo, Norway; 5 Department of Community Medicine, Tromso, Norway; 6 Samfundet Folkhalsan, Helsinki, Finland; 7 Department of Epidemiology, Johns Hopkins Bloomberg School of Public Health, Baltimore, Maryland, United States of America; 8 Brigham and Women's Hospital, Harvard Medical School, Boston, Massachusetts, United States of America; University of Cambridge, United Kingdom

## Abstract

**Background:**

Leisure time physical activity reduces the risk of premature mortality, but the years of life expectancy gained at different levels remains unclear. Our objective was to determine the years of life gained after age 40 associated with various levels of physical activity, both overall and according to body mass index (BMI) groups, in a large pooled analysis.

**Methods and Findings:**

We examined the association of leisure time physical activity with mortality during follow-up in pooled data from six prospective cohort studies in the National Cancer Institute Cohort Consortium, comprising 654,827 individuals, 21–90 y of age. Physical activity was categorized by metabolic equivalent hours per week (MET-h/wk). Life expectancies and years of life gained/lost were calculated using direct adjusted survival curves (for participants 40+ years of age), with 95% confidence intervals (CIs) derived by bootstrap. The study includes a median 10 y of follow-up and 82,465 deaths. A physical activity level of 0.1–3.74 MET-h/wk, equivalent to brisk walking for up to 75 min/wk, was associated with a gain of 1.8 (95% CI: 1.6–2.0) y in life expectancy relative to no leisure time activity (0 MET-h/wk). Higher levels of physical activity were associated with greater gains in life expectancy, with a gain of 4.5 (95% CI: 4.3–4.7) y at the highest level (22.5+ MET-h/wk, equivalent to brisk walking for 450+ min/wk). Substantial gains were also observed in each BMI group. In joint analyses, being active (7.5+ MET-h/wk) and normal weight (BMI 18.5–24.9) was associated with a gain of 7.2 (95% CI: 6.5–7.9) y of life compared to being inactive (0 MET-h/wk) and obese (BMI 35.0+). A limitation was that physical activity and BMI were ascertained by self report.

**Conclusions:**

More leisure time physical activity was associated with longer life expectancy across a range of activity levels and BMI groups.

*Please see later in the article for the Editors' Summary*

## Abstract 2

Analyzing data from over 650,000 individuals, Dr. Steven Moore and colleagues report that greater amounts of leisure-time physical activity were associated with higher life expectancy across a wide range of activity levels and body mass index groups.

## Editors' Summary

**Background:**

Regular physical activity is essential for human health. It helps to maintain a healthy body weight and prevents or delays heart disease, type 2 diabetes, and some cancers. It also makes people feel better and increases life expectancy. The World Health Organization (WHO) currently recommends that adults do at least 150 minutes of moderate- to vigorous-intensity physical activity every week. Moderate-intensity physical activities (for example, brisk walking and gardening) require a moderate amount of effort and noticeably increase the heart rate; vigorous-intensity physical activities (for example, running or fast swimming) require a large amount of effort and cause rapid breathing and a substantial heart rate increase. Worryingly, people in both developed and developing countries are becoming increasingly physically inactive. People are sitting at desks all day instead of doing manual labor; they are driving to work in cars instead of walking or cycling; and they are participating in fewer leisure time physical activities.

**Why Was This Study Done?:**

Although various studies suggest that physical activity increases life expectancy, few have quantified the years of life gained at distinct levels of physical activity. Moreover, the difference in life expectancy between active, overweight individuals and inactive, normal weight individuals has not been quantified. Thus, it is hard to develop a simple public health message to maximize the population benefits of physical activity. In this pooled prospective cohort analysis, the researchers determine the association between levels of leisure time physical activities, such as recreational walking, and years of life gained after age 40, both overall and within body mass index (BMI) groups. A pooled prospective cohort analysis analyzes the combined data from multiple studies that have followed groups of people to investigate associations between baseline characteristics and outcomes such as death. BMI is a ratio of weight to height, calculated by dividing a person's weight by their height squared; normal weight is defined as a BMI of 18.5–24.9 kg/m^2^, obesity (excessive body fat) is defined as a BMI of more than 30 kg/m^2^.

**What Did the Researchers Do and Find?:**

The researchers pooled self-reported data on leisure time physical activities and BMIs from nearly 650,000 individuals over the age of 40 years enrolled in one Swedish and five US prospective cohort studies, most of which were investigating associations between lifestyle factors and disease risk. They used these and other data to calculate the gain in life expectancy associated with specific levels of physical activity. A physical activity level equivalent to brisk walking for up to 75 minutes per week was associated with a gain of 1.8 years in life expectancy relative to no leisure time activity. Being active—having a physical activity level at or above the WHO-recommended minimum of 150 minutes of brisk walking per week—was associated with an overall gain of life expectancy of 3.4–4.5 years. Gains in life expectancy were seen also for black individuals and former smokers, groups for whom relatively few data had been previously available. The physical activity and life expectancy association was also evident at all BMI levels. Being active and normal weight was associated with a gain of 7.2 years of life compared to being inactive and class II+ obese (having a BMI of more than 35.0 kg/m^2^). However, being inactive but normal weight was associated with 3.1 fewer years of life compared to being active but class I obese (having a BMI of 30–34.9 kg/m^2^).

**What Do These Findings Mean?:**

These findings suggest that participation in leisure time physical activity, even below the recommended level, is associated with a reduced risk of mortality compared to participation in no leisure time physical activity. This result may help convince currently inactive people that a modest physical activity program may have health benefits, even if it does not result in weight loss. The findings also suggest that physical activity at recommended levels or higher may increase longevity further, and that a lack of leisure time physical activity may markedly reduce life expectancy when combined with obesity. Although the accuracy and generalizability of these findings may be limited by certain aspects of the study's design (for example, some study participants may have overestimated their leisure time physical activity), these findings reinforce the public health message that both a physically active lifestyle and a normal body weight are important for increasing longevity.

**Additional Information:**

Please access these websites via the online version of this summary at http://dx.doi.org/10.1371/journal.pmed.1001335.

- The World Health Organization provides information about physical activity and health (in several languages); its 2010 Global Recommendations on Physical Activity for Health is available in several languages
- The US Centers for Disease Control and Prevention provides information on physical activity for different age groups; its Physical Activity for Everyone webpages include guidelines, instructional videos, and personal success stories
- The UK National Health Service information source NHS Choices also explains the benefits of regular physical activity and includes physical activity guidelines, tips for exercising, and some personal stories
- MedlinePlus has links to other resources about exercise and physical fitness (in English and Spanish)

## Introduction

In recent decades, physical activity levels have declined in the United States [1] and in other developed and developing nations [2]. Such declines in activity levels could potentially reduce population life expectancy [3]–[5], but this is difficult to quantify because the degree to which the full range of physical activity in different populations is associated with life expectancy remains unclear.

Taken together, more than 100 epidemiologic studies have examined the association between physical activity and risk of mortality. These studies suggest that physical activity has a curvilinear relationship with mortality risk, with a 30% risk reduction among active people during any given period [6]. Few studies, however, have quantified the years of life gained at distinct physical activity levels [7]–[11], and estimates ranged from 2 to 4 y gained with a high versus low level of physical activity. Limitations of these studies include restriction to a single ethnic group [9],[11], small sample size [7],[8],[10], and use of broad physical activity categories [7],[8],[10],[11]. Moreover, they examined different physical activity levels and/or types, complicating efforts to define a straightforward public health message. Finally, there is interest in whether obese and active people live longer than normal weight and inactive people [12], but the difference in life expectancy has not been quantified.

We examined distinct levels of leisure time physical activity of moderate to vigorous intensity in relation to mortality risk and life expectancy in a pooled analysis of six prospective cohort studies including more than 650,000 participants and 82,000 deaths. Our large sample size allowed us to calculate hazard ratios (HRs) of mortality and years of life gained after age 40 according to fine gradations of activity levels and in subgroups for which, to our knowledge, no prior estimates are available, such as black individuals, smokers, and persons with a history of cancer and/or heart disease. We also estimated the association of joint categories of physical activity and body mass index (BMI) with life expectancy.

## Methods

### Inclusion Criteria

Eligible studies were those that participated in a prior analysis of BMI and mortality in the National Cancer Institute Cohort Consortium [13],[14]. Inclusion criteria for the prior analysis were prospective design, five or more years of follow-up, 1,000 or more deaths among non-Hispanic white participants, a study baseline of 1970 or later, and assessment of height, weight, and smoking status. For our analysis, studies were also required to have assessed time spent per week in moderate- and vigorous-intensity leisure time physical activity. If key variables were unavailable at baseline but collected later, baseline was redefined as the later date. Eight of the 19 studies that participated in the BMI and mortality analysis met our criteria, and five agreed to participate. A sixth cohort—the Cancer Prevention Study II—subsequently joined the consortium, met our criteria, and agreed to participate. Participating cohorts were no different from nonparticipating cohorts with respect to prevalence of smoking and co-morbidities, although age at baseline was somewhat higher (average age 7 y higher) for participating cohorts, as was BMI (average BMI 1 kg/m^2^ higher). Each participating study was approved by the institutional review board of the institute that conducted the study.

### Participating Cohorts

The National Institutes of Health (NIH)–AARP Diet and Health Study [15] is a study of diet and health risk factors among members of AARP (formerly known as the American Association of Retired Persons). The Cancer Prevention Study II Nutrition Cohort (CPS II) is a study of environmental and lifestyle risk factors for cancer among US and Puerto Rican men and women [16]. CLUE II is a cardiovascular and cancer risk factor study conducted among Washington County, Maryland residents [17]. The US Radiologic Technologists study is a study of cancer risk factors among radiologic technologists residing in the US and certified by the American Registry of Radiologic Technologists during the period 1926–1982 [18]. The Women's Health Study is a two-by-two factorial clinical trial evaluating low dose aspirin and vitamin E in the prevention of cardiovascular disease and cancer among female health professionals [19]–[21]. The Women's Lifestyle and Health study is a study of oral contraceptives and disease risk among Swedish women sampled from the Uppsala Health Care Region [22].

### Study Variables and Follow-Up

The primary exposure was leisure time physical activity of a moderate or vigorous intensity, which is the intensity level recommended by current guidelines [23]. Leisure time physical activities are those physical activities that are not required as essential activities of daily living and are performed at the discretion of the person [6]. These include activities such as sports, exercising, and recreational walking. Moderate- or vigorous-intensity activities are those with an intensity level of at least three metabolic equivalents (METs) according to the Compendium of Physical Activities [24]. A MET is estimated as the energy cost of a given activity divided by resting energy expenditure [24], and 3 METs (three times resting energy expenditure) is the approximate intensity of a brisk walk. Participants who did no leisure time physical activity of a moderate to vigorous intensity were those who did no exercise or walking outside of the context of a job, housework, transportation, or other essential activities of daily living.

Three studies (CPS II, CLUE II, and Women's Health Study) assessed average time spent per week during the past year performing six or seven leisure time activities (walking, jogging/running, swimming, tennis/racquetball, bicycling, aerobics, and, in CPS II, dance). These questionnaires were adapted from the College Alumni Health Study questionnaire, which had been previously validated (Spearman *r* = 0.67 for leisure time activities by questionnaire versus physical activity level ratio by doubly labeled water) [25]. These three studies comprise 35% of study participants and 52% of study deaths in this analysis.

The physical activity questionnaires in the remaining three studies, while not directly validated, have predicted health outcomes in the expected fashion in prior studies [15],[18],[22]. The NIH-AARP Diet and Health Study assessed hours per week spent in moderate- and/or vigorous-intensity leisure time activities, with elements similar to the validated Physical Activity Scale for the Elderly questionnaire [26]. The Women's Lifestyle and Health study inquired separately about hours per day in moderate- and vigorous-intensity leisure time activities, and the US Radiologic Technologists study asked about hours spent per week in walking for exercise and vigorous exercise. For all six studies, we calculated energy expended per activity by multiplying the MET level [24] by the number of hours per week and summed across activities to estimate overall MET hours per week (MET-h/wk) of leisure time physical activity energy expenditure.

Each study provided baseline data on age, gender, race/ethnicity, highest attained education, smoking status, co-morbidities (cancer except for non-melanoma skin cancer, and heart disease), alcohol consumption, marital status, height, and weight. Variables were classified into standard categories across studies, including race/ethnicity (white, black, other), education (did not complete high school, completed high school, post-high-school training, some college, completed college), smoking status (never, former, current), history of cancer (yes, no), history of heart disease (yes, no), alcohol consumption (0, 0.1–14.9, 15.0–29.9, 30.0+ g/d), marital status (married, divorced, widowed, unmarried), and BMI (<18.5, 18.5–19.9, 20–22.4, 22.5–24.9, 25–27.4, 27.5–29.9, 30+ kg/m^2^). As the proportion of missing data for our covariates was low (6.0% for marital status and <5% for all other covariates), we modeled missing variables using a missing indicator variable. Participants were followed up from baseline to date of death or end of follow-up, whichever was earliest. Date of death was ascertained from death certificates or medical records.

### Statistical Analysis

Proportional hazards regression with stratification by study was used to estimate HRs and 95% confidence intervals (CIs) of mortality for the pooled dataset, with age as the time scale. All analyses were adjusted for gender, alcohol intake, education, marital status, smoking status, and prevalent co-morbidities. Physical activity level was divided into six predefined categories (0, 0.1–3.74, 3.75–7.4, 7.5–14.9, 15.0–22.4, and 22.5+ MET-h/wk) to correspond with cut points in the 2008 US federal physical activity guidelines [23] and the 2010 World Health Organization (WHO) guidelines [27]. These physical activity categories are approximately comparable to brisk walking during leisure time for 0, 1–74, 75–149, 150–299, 300–449, and 450+ min/wk, respectively (MET-h/wk multiplied by 20). Both guidelines recommend a minimum of 150 min/wk of moderate-intensity activity or 75 min/wk of vigorous-intensity physical activity or an equivalent combination (∼7.5 MET-h/wk) for health benefits, and twice that level (∼15.0 MET-h/wk) for additional benefits. We selected 0 MET-h/wk as the referent category.

To assess the dose–response relationship between physical activity and risk of mortality, we used restricted cubic spline models [28]. Linearity of the relationship was evaluated using a likelihood ratio test comparing fit of a spline model selected by a stepwise regression procedure versus fit of a model that included only a linear physical activity term.

In a sensitivity analysis, we further adjusted for prevalent stroke, emphysema, and diabetes, but results were materially the same—the HR for each activity category increased by only approximately 0.02. We also evaluated diet as a potential confounder using the NIH-AARP Diet and Health Study data. We added covariates to the multivariable model—both individually and together—for intake of kilocalories, red meat, fruit (not including juice), vegetables, and multivitamins. These adjustments, however, had no impact on physical activity–mortality associations; all changes in HRs were less than 0.01. In addition to the pooled analysis, we also estimated HRs and 95% CIs using a meta-analysis approach. Meta-analysis estimates were calculated using DerSimonian and Laird random effects models [29], and statistical heterogeneity was assessed by the *I* ^2^ statistic [30].

For each physical activity level, we calculated direct adjusted survival curves [31],[32]. This method uses proportional hazards models to estimate probabilities of survival at each age for each individual and averages them to obtain an overall survival curve. Applying the hazard coefficient for each physical activity level to each individual in the entire study population, survival is then estimated as if assigning all participants alternately to one level of physical activity or another. For each physical activity level, life expectancy was defined as the age of 50% survival. Years of life gained were calculated as the difference between life expectancy for a given physical activity category and that of the reference physical activity category. These models were restricted to participants aged 40 y or more at baseline. For participants with cancer or heart disease, life expectancy models were restricted to participants aged 60 y or more, as these conditions were uncommon in our dataset prior to this age. The variance of the median life expectancies over 100 bootstrap replications was used to calculate 95% CIs under a normal approximation [33].

We assessed interactions of the physical activity and mortality association with gender, race/ethnicity, education level, smoking status, and co-morbidities at baseline. Interactions were declared when the number of years of life gained for the highest physical activity category varied by 0.5 or more years between groups (because of large sample size, tests for interaction were statistically significant even when HRs varied little). The proportional hazards assumption was assessed by examining the physical activity–mortality association according to age group.

We analyzed the combined contribution of physical inactivity and a high BMI to reduced life expectancy using joint categories, where physical activity was classified into three groups (0, 0.1–7.4, 7.5+ MET-h/wk) and BMI was divided into four categories based on WHO clinical cut points [34] for normal weight (BMI 18.5–24.9 kg/m^2^), overweight (BMI 25.0–29.9 kg/m^2^), obese class I (BMI 30.0–34.9 kg/m^2^), and obese class II+ (BMI 35.0+ kg/m^2^). Due to small numbers, underweight participants were omitted from the joint category analysis. The reference category was active (7.5+ MET-h/wk) and normal weight. To address concerns that disease and smoking status may confound the BMI–mortality association [13], we conducted sensitivity analyses restricted to healthy, never smokers.

All statistical analyses were done in SAS, release 9.1, except for random effects meta-analyses, which were done in STATA 10.

## Results

In our pooled dataset, 654,827 out of 687,373 (95.3%) participants had complete data on BMI and physical activity and were used for analyses of physical activity and mortality risk. Of these, 638,855 (97.5%) were aged 40 y or older and were used for the physical activity and life expectancy analyses. After excluding underweight individuals, 632,091 (96.5%) participants remained for the joint physical activity–BMI and life expectancy analyses. During a median 10 y of follow-up, 82,465 deaths were reported (82,315 deaths among individuals aged 40 y or older; 80,767 deaths among individuals aged 40 y or older and also not underweight).

Descriptive statistics for the six cohorts are shown in Table 1. The study population was 56% women and 96.4% white, 2.4% black, and 1.2% of other race/ethnicity. The median age at baseline was 61 y, and the median level of leisure time physical activity of moderate to vigorous intensity was 8 MET-h/wk, with little variation between studies (range: 8–11 MET-h/wk). Participants with higher levels of leisure time physical activity were more likely to be male, non-smokers, college-educated, and not obese (Table 2).

**Table 1:** Selected characteristics according to prospective cohort study.

Study	Total	Males	Females	Study Entry	Median Follow-Up in Years (Maximum)	Median Age at Entry in Years (Range)	Median Physical Activity in MET-h/wk (IQR)	Mean BMI in kg/m^2^ (SD)	Percent Ever Smokers	Percent with Co-Morbidities^a^	Deaths
NIH-AARP Diet and Health Study	312,440	182,598	129,842	1996–1997	10 (10)	64 (51–72)	8 (4–22)	27.0 (4.8)	63	19	36,782
CLUE II	13,661	5,538	8,123	1998	9 (10)	58 (21–98)	9 (4–14)	26.0 (4.7)	43	7	2,049
CPS II	178,402	83,766	94,636	1992–1993	13 (14)	63 (40–91)	8 (4–18)	25.9 (4.3)	56	18	38,776
U.S. Radiologic Technologists study	78,528	18,234	60,294	1994–1998	8 (10)	46 (32–97)	8 (2–22)	25.8 (5.0)	46	8	2,450
Women's Health Study	39,026	0	39,026	1993–1996	13 (15)	53 (39–90)	8 (2–20)	26.0 (5.1)	49	1	2,167
Women's Lifestyle and Health study	32,770	0	32,770	2003	4 (4)	52 (41–62)	11 (4–20)	25.3 (4.2)	57	10	241
Total	654,827	290,136	364,691	1992–2003	10 (15)	61 (21–98)	8 (4–22)	26.4 (4.7)	58	16	82,465

a History of cancer and/or heart disease.

IQR, interquartile range; SD, standard deviation.

**Table 2:** Prevalence of demographic and lifestyle characteristics according to physical activity level.

Characteristic	Category	All Participants	Physical Activity Level (MET-h/wk)					
			0	0.1–3.74	3.75–7.4	7.5–14.9	15.0–22.4	22.5+
**Participants (** ***n*** **)**		654,827	50,555	112,661	60,132	167,931	118,255	145,293
**Age (years)**	<60	287,606	51%	44%	50%	43%	40%	42%
	60–69.9	306,495	38%	45%	43%	47%	52%	49%
	70.0+	60,726	11%	11%	7%	9%	8%	9%
**Gender**	Men	290,136	39%	41%	42%	44%	49%	47%
	Women	364,691	61%	59%	58%	56%	51%	53%
**Smoking status**	Never	272,272	40%	44%	42%	43%	41%	42%
	Former	295,754	41%	43%	44%	46%	49%	48%
	Current	74,341	19%	13%	13%	11%	10%	10%
**Co-morbidities**	Heart disease	60,864	9%	9%	9%	9%	11%	9%
	Cancer	45,894	9%	8%	6%	7%	6%	6%
**Alcohol intake**	0 g/d	178,382	37%	34%	25%	26%	22%	25%
	>0 g/d	476,445	63%	66%	75%	74%	78%	75%
**Education**	High school	155,706	32%	28%	25%	24%	21%	24%
	Some college	221,727	39%	36%	36%	35%	34%	35%
	College graduate	248,783	29%	36%	39%	41%	44%	41%
**Marital status**	Married	469,531	78%	79%	73%	77%	74%	76%
	Not married	145,753	22%	21%	27%	23%	26%	24%
**BMI (kg/m^2^)**	<25.0	280,934	37%	38%	38%	42%	45%	50%
	25.0–29.9	254,704	36%	39%	39%	40%	40%	38%
	30+	119,189	26%	23%	23%	18%	15%	12%
**Race**	White	621,432	96%	96%	95%	97%	96%	97%
	Black	15,455	3%	3%	3%	2%	2%	2%
	Other	7,773	1%	1%	1%	1%	1%	1%

### Leisure Time Physical Activity of a Moderate to Vigorous Intensity and Longevity

**Figure 1:**
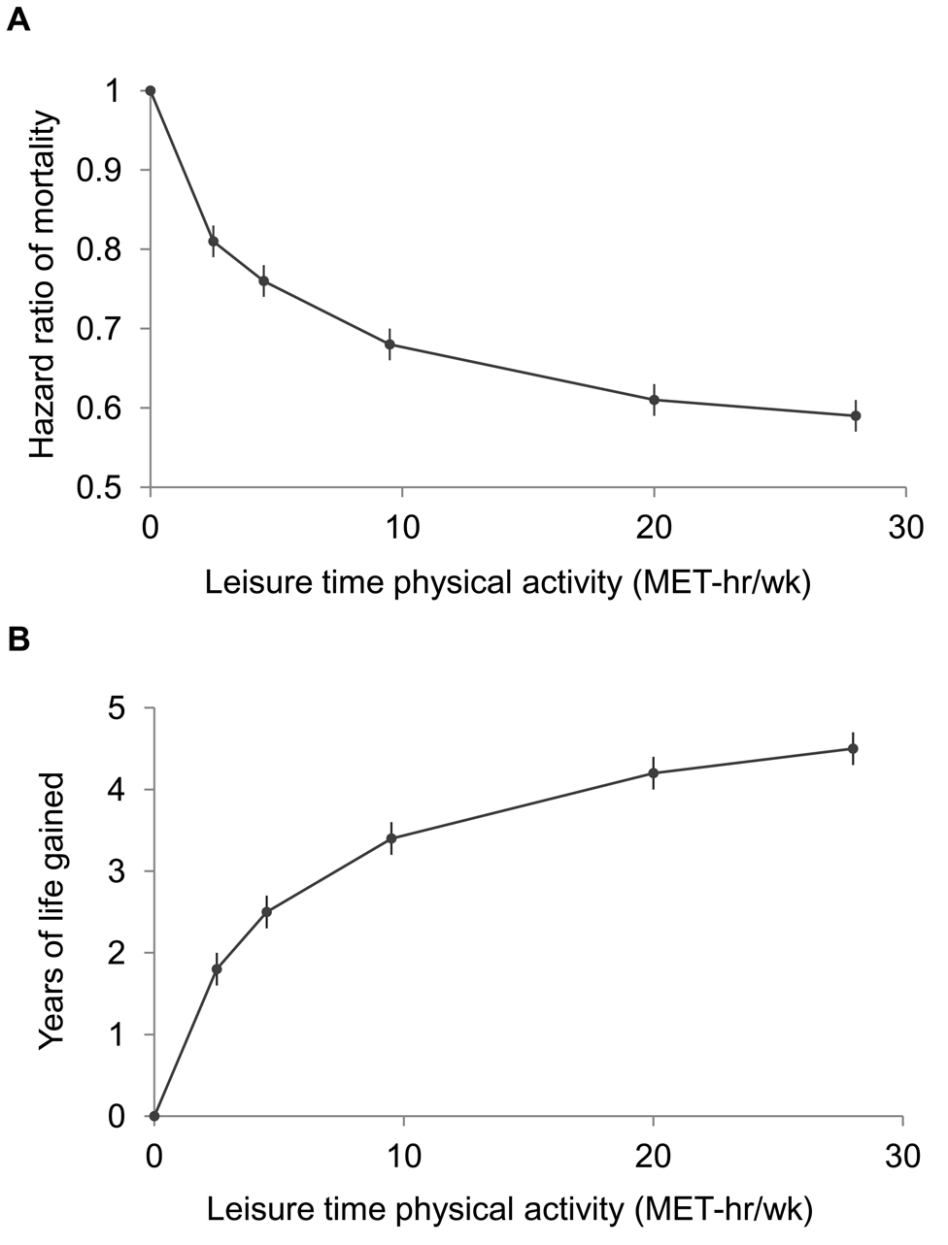
Leisure time physical activity level and hazard ratios for mortality and gains in life expectancy after age 40. The points shown represent the HR (A) or years of life gained (B) for each of the physical activity categories examined, and the vertical lines represent the 95% CIs for that physical activity category. The reference category for both (A) and (B) is 0.0 MET-h/wk of leisure time physical activity. The lines connecting the points help to illustrate the dose–response relationship between physical activity and risk of mortality; the shape of the association shown here is similar to that obtained using spline modeling (Figure S1). HRs were calculated in models stratified by study that used age as the underlying time scale. Multivariable models were adjusted for gender, alcohol consumption (0, 0.1–14.9, 15.0–29.9, 30.0+ g/d), education (did not complete high school, completed high school, post-high-school training, some college, completed college), marital status (married, divorced, widowed, unmarried), history of heart disease, history of cancer, BMI (<18.5, 18.5–19.9, 20–22.4, 22.5–24.9, 25–27.4, 27.5–29.9, 30+ kg/m^2^), and smoking status (never, former, current). Years of life expectancy gained after age 40 were derived using direct adjusted survival curves [31],[32] for participants who were 40+ y of age at baseline (97.5% of participants).

A high level of moderate to vigorous leisure time physical activity was associated with a lower risk of mortality during follow-up and a longer life expectancy after age 40 (Figure 1; Tables 3 and S1). Compared to no leisure time physical activity (0 MET-h/wk), low levels of leisure time physical activity, i.e., 0.1–3.74 MET-h/wk and 3.75–7.4 MET-h/wk, had multivariable-adjusted HRs of 0.81 (95% CI: 0.79–0.83) and 0.76 (0.74–0.78), and life expectancies that were higher by 1.8 (1.6–2.0) and 2.5 (2.2–2.7) y, respectively. Levels at or just above the minimum level recommended by guidelines (7.5–14.9 MET-h/wk) were associated with even lower risks of mortality (HR = 0.68; 95% CI: 0.66–0.69) and higher life expectancies (3.4 y higher; 95% CI: 3.2–3.6). Finally, levels at two times (15.0–22.4 MET-h/wk) and three or more times (22.5+ MET-h/wk) the minimum recommended level were associated with further, albeit diminishing, reductions in risk of mortality. The respective HRs were 0.61 (0.59–0.63) and 0.59 (0.57–0.61), and life expectancies were higher by 4.2 (4.0–4.5) and 4.5 (4.3–4.7) y. Similar results were obtained in models excluding deaths during the first 5 y of follow-up (27,804 deaths excluded; HR for 22.5+ versus 0 MET-h/wk = 0.67; 0.65–0.70). Our spline curves showed that the dose–response relationship was curvilinear (*p*_nonlinear_<0.01), with the greatest gains in years of life expectancy occurring at approximately 15+ MET-h/wk (Figure S1), equivalent to approximately 300 min of brisk walking per week. Trends were similar regardless of age (Table S2), suggesting no violation of the proportional hazards assumption.

**Table 3:** Leisure time physical activity and hazard ratio of mortality and years of life gained after age 40.

Variable	Physical Activity Level (MET-h/wk)					
	0	0.1–3.74	3.75–7.4	7.5–14.9	15.0–22.4	22.5+
**Number of participants**	50,555	112,661	60,132	167,931	118,255	145,293
**Number of deaths**	9,754	18,352	6,968	20,428	11,814	15,149
**Unadjusted HR**	1.0	0.71	0.66	0.56	0.49	0.49
95% CI	Ref	0.70, 0.73	0.64, 0.68	0.55, 0.57	0.48, 0.51	0.48, 0.50
**Gender-adjusted HR**	1.0	0.73	0.67	0.57	0.49	0.48
95% CI	Ref	0.71, 0.75	0.64, 0.69	0.55, 0.58	0.48, 0.51	0.47, 0.50
**Gender-, smoking-adjusted HR**	1.0	0.77	0.70	0.61	0.54	0.53
95% CI	Ref	0.75, 0.79	0.68, 0.73	0.60, 0.63	0.53, 0.56	0.51, 0.54
**Multivariable** a **HR**	1.0	0.81	0.76	0.68	0.61	0.59
95% CI	Ref	0.79, 0.83	0.74, 0.78	0.66, 0.69	0.59, 0.63	0.57, 0.61
**Years of life gained**	—	1.8	2.5	3.4	4.2	4.5
95% CI	Ref	1.6, 2.0	2.2, 2.7	3.2, 3.6	4.0, 4.5	4.3, 4.7

Years of life expectancy gained after age 40 were derived using direct adjusted survival curves [31],[32] for participants who were 40+y of age at baseline (97.5% of participants).

a HRs were calculated in models stratified by study that used age as the underlying time scale. Multivariable models were adjusted for gender, alcohol consumption (0, 0.1–14.9, 15.0–29.9, 30.0+ g/d), education (did not complete high school, completed high school, post-high-school training, some college, completed college), marital status (married, divorced, widowed, unmarried), history of heart disease, history of cancer, BMI (<18.5, 18.5–19.9, 20–22.4, 22.5–24.9, 25–27.4, 27.5–29.9, 30+ kg/m^2^), and smoking status (never, former, current).

The inverse association between physical activity and risk of mortality was evident in all six cohorts, although there was heterogeneity by study (*p*_heterogeneity_<0.05 for all physical activity categories; Table 4; Figures S2, S3, S4, S5, S6). For all but two studies, study-specific HRs were within 0.1 of the pooled estimate. For the Women's Lifestyle and Health study and the Women's Health Study, the HRs for 22.5+ versus 0 MET-h/wk were 0.40 and 0.82, respectively, which differed from the pooled analysis HR of 0.59. However, exclusion of these studies from the analysis did not appreciably change estimates of heterogeneity (Table S3), suggesting that they are not outliers. Moreover, exclusion of these studies—or of any study in the analysis—had little overall influence on the results (Table S4). We further examined the possible impact of heterogeneity by reanalyzing the data using random effects meta-analysis (Table 4). HRs were nearly identical to those of the pooled analysis, and CIs were still tight. There was also some heterogeneity in the associated years of life gained (Table S5), with estimates ranging from 1.1 to 6.0 y of life gained for 22.5+ versus 0 MET-h/wk, primarily reflecting differences in HRs between studies.

**Table 4:** Leisure time physical activity and multivariable hazard ratio of mortality, stratified by cohort.

Study	Number of Deaths	Physical Activity Level (MET-h/wk)					
		0	0.1–3.74	3.75–7.4	7.5–14.9	15.0–22.4	22.5+
AARP	36,782	1.0 (ref)	0.80 (0.76, 0.84)	0.68 (0.65, 0.72)	0.59 (0.57, 0.62)	0.52 (0.50, 0.55)	0.48 (0.45, 0.50)
CLUE II	2,049	1.0 (ref)	0.73 (0.63, 0.84)	0.62 (0.54, 0.71)	0.57 (0.50, 0.65)	0.61 (0.50, 0.74)	0.52 (0.42, 0.64)
CPS II	38,776	1.0 (ref)	0.80 (0.77, 0.83)	0.79 (0.74, 0.83)	0.70 (0.68, 0.72)	0.65 (0.62, 0.68)	0.65 (0.63, 0.67)
USRT	2,450	1.0 (ref)	0.71 (0.63, 0.80)	0.54 (0.45, 0.66)	0.57 (0.51, 0.64)	0.54 (0.46, 0.63)	0.55 (0.49, 0.62)
WHS	2,167	1.0 (ref)	0.87 (0.76, 0.99)	0.79 (0.67, 0.92)	0.70 (0.60, 0.81)	0.80 (0.68, 0.94)	0.82 (0.70, 0.94)
WLH	241	1.0 (ref)	0.44 (0.29, 0.68)	0.36 (0.23, 0.58)	0.30 (0.19, 0.45)	0.35 (0.22, 0.56)	0.40 (0.27, 0.59)
Pooled	82,465	1.0 (ref)	0.81 (0.79, 0.83)	0.76 (0.74, 0.78)	0.68 (0.66, 0.69)	0.61 (0.59, 0.63)	0.59 (0.57, 0.61)
Meta-analysis^a^	82,465	1.0 (ref)	0.78 (0.73, 0.83)	0.66 (0.59, 0.75)	0.60 (0.53, 0.67)	0.59 (0.51, 0.68)	0.57 (0.48, 0.68)
*I* ^2^ (*p*-value)	—	—	64.6% (0.02)	86.7% (<0.01)	92.6% (<0.01)	92.1% (<0.01)	95.8% (<0.01)

HRs (95% CIs) were calculated in models that used age as the underlying time scale. Multivariable models were adjusted for gender, alcohol consumption (0, 0.1–14.9, 15.0–29.9, 30.0+ g/d), education (did not complete high school, completed high school, post-high-school training, some college, completed college), marital status (married, divorced, widowed, unmarried), history of heart disease, history of cancer, BMI (<18.5, 18.5–19.9, 20–22.4, 22.5–24.9, 25–27.4, 27.5–29.9, 30+ kg/m^2^), and smoking status (never, former, current).

a Meta-analysis estimates were calculated using DerSimonian and Laird random effects models [29], and statistical heterogeneity was assessed by the *I* ^2^ statistic [30].

AARP, NIH-AARP Diet and Health Study; ref, reference; USRT, U.S. Radiologic Technologists cohort; WHS, Women's Health Study; WLH, Women's Lifestyle and Health study.

We further examined whether the association between physical activity and life expectancy varied according to gender, race/ethnicity, and education (Table 5). The association was similar between men and women; a leisure time physical activity level of 22.5+ versus 0 MET-h/wk was associated with 4.7 (95% CI: 4.4–4.9) more years of life among men and 4.5 (4.1–5.0) more years of life among women. For other subgroups, there was evidence of interaction. The association between physical activity (22.5+ versus 0 MET-h/wk) and higher life expectancy was more robust among black individuals (6.4 y; 4.2–8.5) than among white individuals (4.5 y; 4.4–4.7) and among participants with some college or post-high-school training (5.1 y; 4.7–5.5) than among those with only a high school education (4.3 y; 4.0–4.6) or a college or graduate degree (4.5 y; 4.1–4.9).

**Table 5:** Leisure time physical activity and multivariable hazard ratio of mortality and years of life gained after age 40 for all participants and according to gender and race/ethnicity.

Variable	Physical Activity Level (MET-h/wk)					
	0	0.1–3.74	3.75–7.4	7.5–14.9	15.0–22.4	22.5+
***Gender***						
**Men**						
Number of deaths	5,767	10,975	4,241	12,713	7,443	10,033
Multivariable HR	1.0	0.84	0.77	0.69	0.60	0.59
95% CI	Ref	0.81, 0.87	0.74, 0.80	0.67, 0.71	0.58, 0.63	0.57, 0.61
Years of life gained	—	1.6	2.3	3.3	4.5	4.7
95% CI	Ref	1.4, 1.8	2.0, 2.6	3.1, 3.5	4.3, 4.7	4.4, 4.9
**Women**						
Number of deaths	3,987	7,377	2,727	7,715	4,371	5,116
Multivariable HR	1.0	0.76	0.71	0.63	0.60	0.56
95% CI	Ref	0.73, 0.79	0.68, 0.75	0.61, 0.66	0.57, 0.62	0.54, 0.59
Years of life gained	—	2.1	2.7	3.6	4.0	4.5
95% CI	Ref	1.7, 2.5	2.3, 3.1	3.1, 4.0	3.4, 4.6	4.1, 5.0
***Race/ethnicity***						
**White (European descent)**						
Number of deaths	9,334	17,535	6,543	19,592	11,168	14,520
Multivariable HR	1.0	0.81	0.76	0.68	0.61	0.59
95% CI	Ref	0.79, 0.83	0.74, 0.79	0.66, 0.70	0.59, 0.63	0.58, 0.61
Years of life gained	—	1.9	2.4	3.4	4.3	4.5
95% CI	Ref	1.7, 2.1	2.2, 2.6	3.2, 3.6	4.1, 4.6	4.3, 4.7
**Black (African descent)**						
Number of deaths	260	442	217	386	298	255
Multivariable HR	1.0	0.83	0.71	0.61	0.71	0.54
95% CI	Ref	0.71, 0.97	0.59, 0.85	0.52, 0.71	0.60, 0.84	0.46, 0.65
Years of life gained	—	2.6	3.7	5.3	3.6	6.4
95% CI	Ref	1.2, 4.1	2.0, 5.3	3.5, 7.1	2.0, 5.1	4.2, 8.5
***Education***						
**High school or less**						
Number of deaths	3,985	6,311	2,116	5,777	3,061	4,353
Multivariable HR	1.0	0.84	0.76	0.69	0.65	0.62
95% CI	Ref	0.81, 0.87	0.72, 0.80	0.66, 0.72	0.62, 0.69	0.59, 0.65
Years of life gained	—	1.7	2.5	3.4	3.9	4.3
95% CI	Ref	1.4, 2.0	2.0, 3.0	3.0, 3.7	3.6, 4.3	4.0, 4.6
**Some college or post-high-school training**						
Number of deaths	3,040	5,988	2,429	6,836	3,986	4,925
Multivariable HR	1.0	0.79	0.74	0.66	0.60	0.57
95% CI	Ref	0.75, 0.82	0.70, 0.78	0.64, 0.69	0.57, 0.63	0.54, 0.59
Years of life gained	—	2.2	2.8	3.7	4.7	5.1
95% CI	Ref	1.8, 2.6	2.5, 3.2	3.3, 4.1	4.3, 5.1	4.7, 5.5
**College graduate**						
Number of deaths	2,294	5,644	2,238	7,332	4,425	5,440
Multivariable HR	1.0	0.80	0.78	0.67	0.60	0.59
95% CI	Ref	0.76, 0.84	0.74, 0.83	0.64, 0.71	0.57, 0.63	0.56, 0.62
Years of life gained	—	1.9	2.1	3.3	4.4	4.5
95% CI	Ref	1.5, 2.2	1.6, 2.5	2.9, 3.7	4.0, 4.8	4.1, 4.9

HRs were calculated in models stratified by study that used age as the underlying time scale. Multivariable models were adjusted for gender, alcohol consumption (0, 0.1–14.9, 15.0–29.9, 30.0+ g/d), education (did not complete high school, completed high school, post-high-school training, some college, completed college), marital status (married, divorced, widowed, unmarried), history of heart disease, history of cancer, BMI (<18.5, 18.5–19.9, 20–22.4, 22.5–24.9, 25–27.4, 27.5–29.9, 30+ kg/m^2^), and smoking status (never, former, current). If a covariate was a stratification variable for a particular model, then it was excluded from multivariable adjustment. Years of life expectancy gained after age 40 were derived using direct adjusted survival curves [31],[32] for participants who were 40+y of age at baseline (97.5% of participants).

Similarly, the association between physical activity (22.5+ versus 0 MET-h/wk) and higher life expectancy was stronger among former smokers (5.5 y; 5.2–5.7) than among never smokers (3.3 y; 3.0–3.7) or current smokers (3.5 y; 3.0–3.9), as well as among those with a history of cancer (7.0 y; 6.5–7.5) or heart disease (6.2 y; 5.7–6.7) than among those without such history (3.7 y; 3.5–3.9) (Table 6). For the healthiest group—never smokers with no history of heart disease or cancer—a high physical activity level was associated with 2.8 (2.4–3.2) more years of life expectancy, which is a weaker association than in other groups but still robust.

**Table 6:** Leisure time physical activity and multivariable hazard ratio of mortality and years of life gained after age 40 according to smoking and co-morbidity status.

Variable	Physical Activity Level (MET-h/wk)					
	0	0.1–3.74	3.75–7.4	7.5–14.9	15.0–22.4	22.5+
***Smoking status***						
**Never smoker**						
Number of deaths	2,521	5,430	1,814	5,857	3,200	4,283
Multivariable HR	1.0	0.80	0.75	0.67	0.64	0.62
95% CI	Ref	0.76, 0.84	0.70, 0.79	0.63, 0.70	0.61, 0.68	0.59, 0.65
Years of life gained	—	1.6	2.2	3.0	3.2	3.3
95% CI	Ref	1.2, 1.9	1.6, 2.8	2.7, 3.3	2.8, 3.6	3.0, 3.7
**Former smoker**						
Number of deaths	4,876	9,306	3,617	10,962	6,465	8,107
Multivariable HR	1.0	0.77	0.72	0.63	0.55	0.52
95% CI	Ref	0.74, 0.80	0.69, 0.75	0.61, 0.65	0.53, 0.57	0.50, 0.54
Years of life gained	—	2.2	2.8	4.1	5.0	5.5
95% CI	Ref	2.0, 2.5	2.3, 3.3	3.8, 4.3	4.8, 5.2	5.2, 5.7
**Current smoker**						
Number of deaths	2,187	3,279	1,335	3,187	1,846	2,403
Multivariable HR	1.0	0.86	0.76	0.75	0.67	0.68
95% CI	Ref	0.82, 0.91	0.70, 0.81	0.71, 0.79	0.62, 0.71	0.64, 0.72
Years of life gained	—	1.3	2.5	2.6	3.7	3.5
95% CI	Ref	0.9, 1.7	1.9, 3.1	2.1, 3.0	3.2, 4.2	3.0, 3.9
***Pre-existing co-morbidity*** a						
**Cancer**						
Number of deaths	1,765	3,324	963	3,268	1,603	2,217
Multivariable HR	1.0	0.81	0.74	0.65	0.59	0.55
95% CI	Ref	0.76, 0.86	0.68, 0.80	0.62, 0.69	0.55, 0.63	0.52, 0.59
Years of life gained	—	2.7	3.6	5.3	6.2	7.0
95% CI	Ref	2.2, 3.2	2.7, 4.4	4.9, 5.7	5.7, 6.7	6.5, 7.5
**Heart disease**						
Number of deaths	1,884	3,521	1,531	4,125	2,550	2,929
Multivariable HR	1.0	0.79	0.72	0.61	0.51	0.51
95% CI	Ref	0.74, 0.83	0.67, 0.77	0.58, 0.65	0.48, 0.54	0.48, 0.54
Years of life gained	—	2.0	2.8	4.3	5.9	6.2
95% CI	Ref	1.5, 2.5	2.3, 3.4	3.9, 4.7	5.4, 6.4	5.7, 6.7
**None**						
Number of deaths	6,451	12,029	4,668	13,584	7,961	10,380
Multivariable HR	1.0	0.80	0.75	0.68	0.63	0.61
95% CI	Ref	0.78, 0.83	0.72, 0.78	0.66, 0.70	0.60, 0.65	0.59, 0.62
Years of life gained	—	1.6	2.0	2.8	3.4	3.7
95% CI	Ref	1.4, 1.8	1.8, 2.3	2.5, 3.0	3.2, 3.7	3.5, 3.9
**Healthy never smokers** b						
Number of deaths	1,762	3,796	1,312	4,118	2,272	3,104
Multivariable HR	1.0	0.79	0.75	0.68	0.67	0.65
95% CI	Ref	0.75, 0.84	0.70, 0.91	0.64, 0.72	0.62, 0.71	0.61, 0.69
Years of life gained	—	1.4	1.8	2.6	2.7	2.8
95% CI	Ref	0.9, 1.9	1.3, 2.3	2.2, 3.0	2.2, 3.2	2.3, 3.3

HRs were calculated in models stratified by study that used age as the underlying time scale. Multivariable models were adjusted for gender, alcohol consumption (0, 0.1–14.9, 15.0–29.9, 30.0+ g/d), education (did not complete high school, completed high school, post-high-school training, some college, completed college), marital status (married, divorced, widowed, unmarried), history of heart disease, history of cancer, BMI (<18.5, 18.5–19.9, 20–22.4, 22.5–24.9, 25–27.4, 27.5–29.9, 30+ kg/m^2^), and smoking status (never, former, current). If a covariate was a stratification variable for a particular model, then it was excluded from multivariable adjustment. Years of life expectancy gained after age 40 were derived using direct adjusted survival curves [31],[32] for participants who were 40+ y of age at baseline (97.5% of participants).

a Years of life expectancy gained after age 60. Cancer and/or heart disease were uncommon prior to this age in our dataset.

b Participants who had never smoked and who had no history of heart disease or cancer.

### Leisure Time Physical Activity of Moderate to Vigorous Intensity, BMI, and Their Joint Relation to Longevity

**Figure 2:**
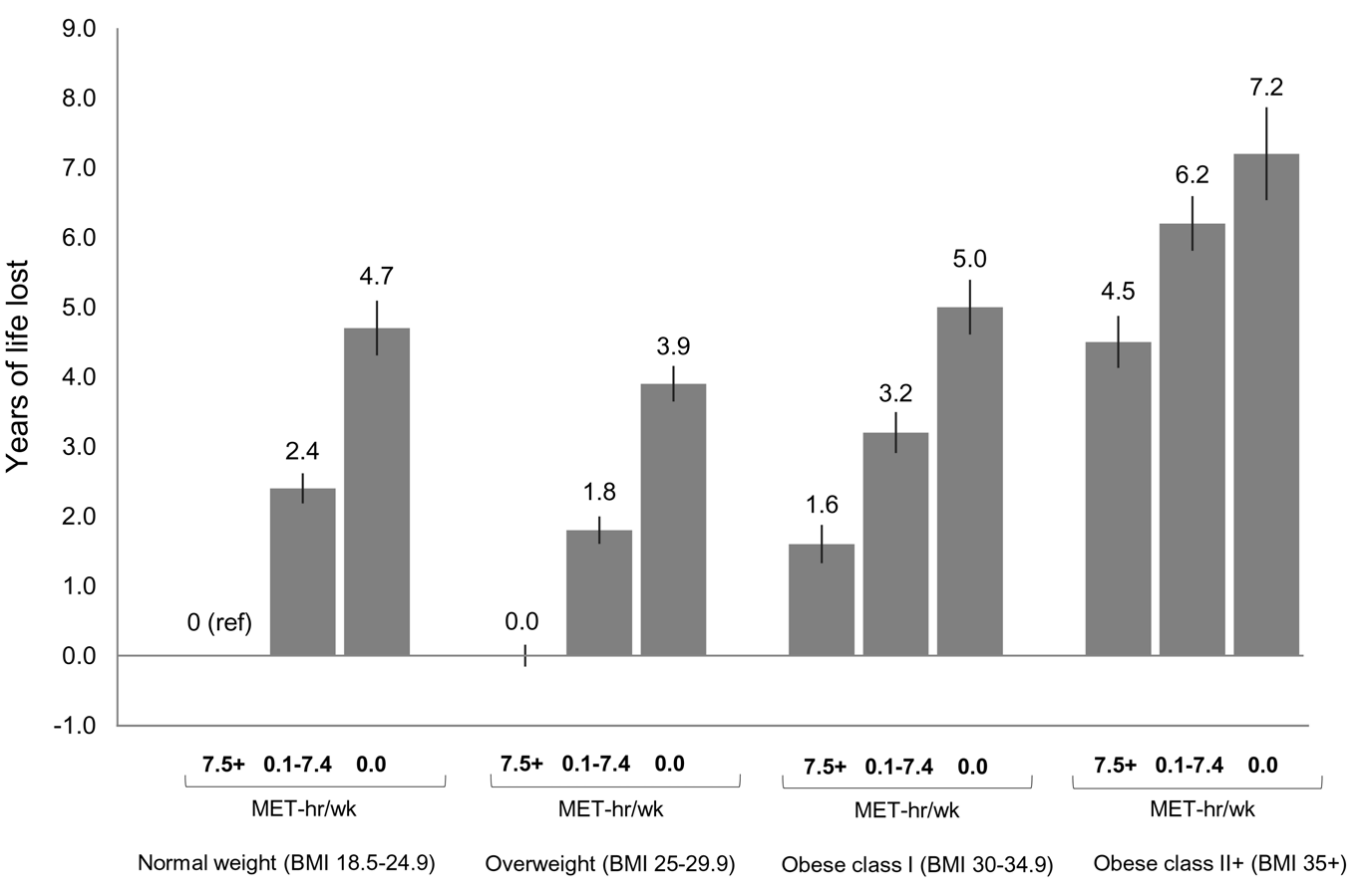
Years of life expectancy lost after age 40 in relation to joint categories of physical activity level and body mass index. The bars indicate the number of years of life lost for each category, and the vertical lines are the 95% CIs. The reference category is normal weight and 7.5+ MET-h/wk of physical activity (i.e., meeting US recommended physical activity levels). Normal weight is a BMI of 18.5–24.9 kg/m^2^, overweight is a BMI of 25.0–29.9 kg/m^2^, obese class I is a BMI of 30.0–34.9 kg/m^2^, and obese class II+ is a BMI of 35.0 kg/m^2^ or greater. Years of life expectancy lost after age 40 were derived using direct adjusted survival curves [31],[32] for participants who were 40+ y of age at baseline and not underweight (96.5% of participants). Life expectancy models used age as the underlying time scale and were adjusted for gender, alcohol consumption (0, 0.1–14.9, 15.0–29.9, 30.0+ g/d), education (did not complete high school, completed high school, post-high-school training, some college, completed college), marital status (married, divorced, widowed, unmarried), history of heart disease, history of cancer, and smoking status (never, former, current).

In analyses of joint categories, we found that low physical activity was associated with lower life expectancy and greater risk of death in each BMI group (Figures 2 and S7). For example, among normal weight participants, being inactive (0 MET-h/wk of leisure time physical activity) was associated with 4.7 fewer years of life versus being active (7.5+ MET-h/wk). The same contrast (0 versus 7.5+ MET-h/wk) was associated with 3.9 fewer years of life among overweight participants, 3.4 fewer years among obese class I participants, and 2.7 fewer years among obese class II participants.

Likewise, we found that obesity (but not overweight) was associated with lower life expectancy in each physical activity group. For example, among low activity (0.1–7.4 MET-h/wk) participants, overweight, obese class I, and obese class II+ were associated with −0.6, 0.8, and 3.8 fewer years of life, respectively, compared to normal weight. Among active participants, the same respective BMI categories were associated with 0.0, 1.6, and 4.5 fewer years of life, compared to normal weight. Among active participants who were healthy and never smoked (Figures S7 and S8), these BMI categories were associated with 0.5, 2.5, and 5.2 fewer years of life, compared to normal weight. Among healthy never smokers, overweight was generally associated with lower life expectancy than normal weight (Figure S9), but results in this group were otherwise similar in substance to those for the total study population.

We also examined which joint category was associated with shorter life expectancy: normal weight–inactive or obese–active (Figure 2). Compared to the obese class I–active category, the normal weight–inactive category was associated with 3.1 fewer years of life, and the normal weight–low active category was associated with 0.8 fewer years of life. However, compared to the obese class II+–active category, there was no significant difference in life expectancy for the normal weight–inactive category, and the normal weight–low active category was associated with 2.1 *more* years of life.

At the extremes, the obese class II+–inactive group was associated with 7.2 (6.5,7.9) fewer years of life than the normal weight–active group (reference group).

## Discussion

In this large pooled analysis, participation in even a low level of leisure time physical activity of moderate to vigorous intensity—i.e., 0.1–3.74 MET-h/wk, equivalent to less than half the WHO-recommended activity level and comparable to up to 75 min of brisk walking per week—was associated with reduced risk of mortality during follow-up relative to no such activity. Assuming a causal relationship, this level of activity would confer a 1.8-y gain in life expectancy after age 40, compared with no activity. At the minimum recommended physical activity level—7.5–14.9 MET-h/wk, equivalent to 150–299 min of brisk walking per week—the gain in life expectancy was 3.4 y. At approximately two times the minimum recommended level—15.0–22.4 MET-h/wk, which is equivalent to brisk walking for 300–449 min/wk—the gain in life expectancy was 4.2 y. The association between physical activity and life expectancy was evident at every level of BMI. Combined together, a lack of activity and a high BMI (obese class II+) were associated with 7.2 y of life lost relative to meeting recommended activity levels and being normal weight. For comparison, long-term cigarette smoking reduces life expectancy by approximately 10 y [35]. Our findings highlight the important contribution of physical activity to longevity.

The dose–response relationship that we observed between physical activity and mortality is consistent with the findings of the Physical Activity Guidelines Advisory Committee report [6], a report that formed the basis for the 2008 US federal physical activity guidelines [23] and helped inform the WHO physical activity guidelines [27]. The Physical Activity Guidelines Advisory Committee report reviewed all published studies through 2008 to estimate the physical activity and mortality dose–response relationship and preliminarily reported that a low physical activity level was associated with a 20% reduced risk of mortality relative to no leisure time activity [6]. The authors cautioned, however, that additional data were needed to confirm this estimate. Our study, which included 82,000+ deaths—more than four times the 18,500 deaths in the committee's review and with only 241 deaths overlapping between the report and this study [6]—provides such corroborating data. The comparable low level of activity in our study was associated with a 19% lower risk of mortality, nearly identical to the committee's estimate. The committee also reported that the overall dose–response relationship was curvilinear, with diminishing returns at high activity levels, and our results show the same pattern. Finally, the maximum reduction in risk of mortality in the committee's analysis was 39%, and ours was an almost identical 41%. Our results substantiate those of the Physical Activity Guidelines Advisory Committee analysis.

Going beyond the Physical Activity Guidelines Advisory Committee report, the large sample size of our study allowed us to address the report's recommendation that future studies should “examine interactions between sociodemographic factors, particularly sex and race/ethnicity or SES [socioeconomic status], and physical activity in relation to health” [6]. In our study, the longevity benefits of physical activity did not vary by gender, similar to prior reports [9], but did vary by educational status, race/ethnicity, smoking status, and especially by pre-existing co-morbidity. These results support the benefit of physical activity for all these subgroups, although results for pre-existing co-morbidities should be interpreted with caution. Severe disease is associated with risk of mortality and also, in some cases, with reduced physical activity. This could confound associations and cause the benefits of physical activity to be overstated.

The years of life gained associated with physical activity in our study were within the range of those in prior publications [7]–[9]. The Harvard Alumni study examined an index comprising outdoor walking, stair climbing, and sports (predominantly leisure activities) and found that an expenditure of 2,000+ versus 500 or fewer kilocalories per week was associated with a 2.3-y gain in life expectancy [7]. For individuals weighing 68 kg (150 pounds), this is like comparing 29+ versus 7 or fewer MET-h/wk—a contrast associated with 2–3 y of life gained in our study. Recently, a study in Taiwan [9] found that leisure time physical activity at or above the minimum recommended level (7.5+ MET-h/wk) was associated with a gain of 3.7–4.2 y of life expectancy versus inactivity (<3.75 MET-h/wk). This contrast would be associated with 4 y of life gained in our study. The similarity of our results and theirs suggests that the longevity benefits of physical activity are transnational and cross-cultural.

Other studies reported activity levels using different metrics than our own, but obtained broadly consistent estimates. The Seventh Day Adventist study [11] assessed an index comprising vigorous exercise and occupational activity, and found that a high versus low level of activity was associated with 1.9–2.7 y of life gained. The Framingham Heart Study [8] examined total energy expenditure, including energy expended while sleeping or sedentary, and found that an expenditure of 33+ versus 30 or fewer MET-h/wk was associated with 3.5–3.7 y of life gained. The Uppsala Longitudinal Study [10] ascertained leisure time physical activity, and a high versus low activity level was associated with 3.8 y of life gained.

To our knowledge, our study is the first to estimate years of life lost for joint categories of physical inactivity and BMI. Earlier studies examined risk of mortality as opposed to years of life lost. The first studies in this area reported that low fitness was of far greater consequence to mortality risk than was obesity [12],[36]. Subsequent studies examined physical inactivity, rather than fitness [12],[15], and found weaker results, but there was still a suggestion that obese and active people may live longer than normal weight and inactive people. We examined this hypothesis and found that class I obese and active participants lived longer than normal weight and inactive participants. Class II+ obese and active participants, however, did not live longer.

The strengths of our study include a large study population with a broad age range, a reasonably large number of black participants, and the use of uniform physical activity categories matched to levels described in current guidelines. In addition, we were able to estimate survival curves directly from our own data, allowing us to calculate years of life gained for subgroups not previously investigated.

The primary limitation of the study is its reliance upon self-reported leisure time physical activity. In prospective studies, errors in recall of physical activity are likely non-differential with respect to disease, resulting in attenuated associations [37]–[39], although research is ongoing. It is likely that overweight and obese participants overreport leisure time physical activity [40], which could attenuate physical activity–mortality associations in these groups. Future studies may be able to quantify these errors and correct associations using measurement error correction models, as long as reference measures appropriately match the scope and types of activities assessed by the questionnaire [39]. An additional limitation of our analysis is that it focused on leisure time physical activity of a moderate to vigorous intensity: other types and intensities of physical activity [41] and sedentary behaviors [42] may also contribute to mortality risk.

Errors in recalled height and weight could cause some overstatement of the harms of obesity [43], although this effect would likely be modest [13]. Typically, participants overestimate height and underestimate weight, resulting in a BMI that is, on average, 1 kg/m^2^ lower than when height and weight are directly measured [43]. BMI is also limited in that it does not discriminate between fat and lean body mass. However, validation studies show that correlations between BMI and body fat percent are high [44].

Due to the observational study design, we cannot fully exclude the possibility of residual confounding by co-morbidities, diet, or other factors. Our analysis adjusted for heart disease and cancer, and, in sensitivity analyses, for stroke, emphysema, and diabetes, with minimal overall impact on HRs. Nonetheless, we lacked detailed information on other co-morbidities, and these other co-morbidities could confound associations to some degree. We did not adjust for diet in our analysis. However, adjustment for dietary factors in a sensitivity analysis had little impact on results, suggesting against major confounding by diet. Possibly, potential confounding by diet is tempered by our adjustment for covariates related to diet, such as age and gender.

An additional limitation is that our pooled studies included somewhat different populations and somewhat different assessments of physical activity. This likely contributed to the heterogeneity that we observed between studies in the physical activity and mortality association. Nevertheless, all of the studies showed an inverse association between physical activity and mortality, and the magnitude of the associations was mostly similar between studies. Finally, our analysis was based primarily on white people from developed nations, which may limit generalizability. Results to date suggest that the physical activity–mortality association is similar across racial/ethnic groups and across nations [9],[41], but more research is needed to determine this definitively.

In conclusion, adding even low amounts of leisure time physical activity to one's daily routine—such as 75 min of walking per week—may increase longevity. This finding may help convince currently inactive persons that a modest physical activity program is “worth it” for health benefits, even if it may not result in weight control [6]. Physical activity above the minimal level—at recommended levels, or even higher—appears to increase longevity even further, with the increase in longevity starting to plateau at approximately 300 min of brisk walking per week. Finally, a lack of leisure time physical activity when combined with obesity is associated with markedly diminished life expectancy. Together, these findings reinforce prevailing public health messages and support them for a range of ages and backgrounds: both a physically active lifestyle and a normal body weight are important for increasing longevity.

## Supporting Information

Figure S1 **Leisure time physical activity and hazard ratios of mortality.** In this figure, the line is a natural cubic spline [28] showing the shape of the dose–response curve for the association between physical activity and risk of mortality. HRs are indicated by the solid line, and 95% CIs by the dashed lines. The reference point is 0.0 MET-h/wk, with knots at 0, 7.5, 21.6, and 30.3 MET-h/wk. The graphic display is truncated at 30.0 MET-h/wk, which is the 95th percentile of the physical activity distribution. All models are adjusted for gender, alcohol consumption (0, 0.1–14.9, 15.0–29.9, 30.0+ g/d), education (did not complete high school, completed high school, post-high-school training, some college, completed college), marital status (married, divorced, widowed, unmarried), history of heart disease, history of cancer, BMI (<18.5, 1.8–19.9, 20–22.4, 22.5–24.9, 25–27.4, 27.5–29.9, 30+ kg/m^2^), and smoking status (never, former, current).
(TIF)

Figure S2 **Forest plot of association between leisure time physical activity and mortality: 0.1–3.74 MET-h/wk versus 0.0 MET-h/wk.** HRs are indicated by the box, and the size of the box is inversely proportional to the variance of the log HR estimate in each cohort. The lines show the 95% CIs. Models are adjusted for gender, alcohol consumption (0, 0.1–14.9, 15.0–29.9, 30.0+ g/d), education (did not complete high school, completed high school, post-high-school training, some college, completed college), marital status (married, divorced, widowed, unmarried), history of heart disease, history of cancer, BMI (<18.5, 1.8–19.9, 20–22.4, 22.5–24.9, 25–27.4, 27.5–29.9, 30+ kg/m^2^), and smoking status (never, former, current).
(TIF)

Figure S3 **Forest plot of association between leisure time physical activity and mortality: 3.75–7.4 MET-h/wk versus 0.0 MET-h/wk.** HRs are indicated by the box, and the size of the box is inversely proportional to the variance of the log HR estimate in each cohort. The lines show the 95% CIs. Models are adjusted for gender, alcohol consumption (0, 0.1–14.9, 15.0–29.9, 30.0+ g/d), education (did not complete high school, completed high school, post-high-school training, some college, completed college), marital status (married, divorced, widowed, unmarried), history of heart disease, history of cancer, BMI (<18.5, 1.8–19.9, 20–22.4, 22.5–24.9, 25–27.4, 27.5–29.9, 30+ kg/m^2^), and smoking status (never, former, current).
(TIF)

Figure S4 **Forest plot of association between leisure time physical activity and mortality: 7.5–14.9 MET-h/wk versus 0.0 MET-h/wk.** HRs are indicated by the box, and the size of the box is inversely proportional to the variance of the log HR estimate in each cohort. The lines show the 95% CIs. Models are adjusted for gender, alcohol consumption (0, 0.1–14.9, 15.0–29.9, 30.0+ g/d), education (did not complete high school, completed high school, post-high-school training, some college, completed college), marital status (married, divorced, widowed, unmarried), history of heart disease, history of cancer, BMI (<18.5, 1.8–19.9, 20–22.4, 22.5–24.9, 25–27.4, 27.5–29.9, 30+ kg/m^2^), and smoking status (never, former, current).
(TIF)

Figure S5 **Forest plot of association between leisure time physical activity and mortality: 15.0–22.4 MET-h/wk versus 0.0 MET-h/wk.** HRs are indicated by the box, and the size of the box is inversely proportional to the variance of the log HR estimate in each cohort. The lines show the 95% CIs. Models are adjusted for gender, alcohol consumption (0, 0.1–14.9, 15.0–29.9, 30.0+g/d), education (did not complete high school, completed high school, post-high-school training, some college, completed college), marital status (married, divorced, widowed, unmarried), history of heart disease, history of cancer, BMI (<18.5, 1.8–19.9, 20–22.4, 22.5–24.9, 25–27.4, 27.5–29.9, 30+kg/m^2^), and smoking status (never, former, current).
(TIF)

Figure S6 **Forest plot of association between leisure time physical activity and mortality: 22.5+ MET-h/wk versus 0.0 MET-h/wk.** HRs are indicated by the box, and the size of the box is inversely proportional to the variance of the log HR estimate in each cohort. The lines show the 95% CIs. Models are adjusted for gender, alcohol consumption (0, 0.1–14.9, 15.0–29.9, 30.0+g/d), education (did not complete high school, completed high school, post-high-school training, some college, completed college), marital status (married, divorced, widowed, unmarried), history of heart disease, history of cancer, BMI (<18.5, 1.8–19.9, 20–22.4, 22.5–24.9, 25–27.4, 27.5–29.9, 30+kg/m^2^), and smoking status (never, former, current).
(TIF)

Figure S7 **Hazard ratios of mortality in relation to joint categories of physical activity level and body mass index.** HRs are shown for participants aged 40 y or older who had a BMI of at least 18.5 kg/m^2^ (*n* = 632,091; deaths = 80,767). The bars indicate the HRs for each joint category, and the vertical lines are the 95% CIs. The reference category is normal weight and 7.5+ MET-h/wk of physical activity (i.e., meeting US recommended physical activity levels). The multivariable HRs were calculated in models stratified by study that used age as the underlying time metric. Models were adjusted for gender, alcohol consumption (0, 0.1–14.9, 15.0–29.9, 30.0+ g/d), education (did not complete high school, completed high school, post-high-school training, some college, completed college), marital status (married, divorced, widowed, unmarried), history of heart disease, history of cancer, BMI (<18.5, 18.5–19.9, 20–22.4, 22.5–24.9, 25–27.4, 27.5–29.9, 30+ kg/m^2^), and smoking status (never, former, current).
(TIF)

Figure S8 **Years of life expectancy lost after age 40 in relation to joint categories of physical activity level and body mass index among healthy never smokers.** Years of life expectancy gained/lost according to level of physical activity and BMI are shown for participants aged 40 y or older with no history of smoking, heart disease, or cancer and with a BMI of at least 18.5 kg/m^2^ (*n* = 236,828; deaths = 16,074). The bars indicate the number of years of life lost for each category, and the vertical lines are the 95% CIs. The reference category is normal weight and 7.5+ MET-h/wk of physical activity (i.e., meeting US recommended physical activity levels). Years of life expectancy lost after age 40 were derived using direct adjusted survival curves [31],[32] for participants who were 40+ y of age at baseline. By applying the hazard coefficient for each joint category to the entire study population, survival is estimated as if assigning all participants alternately to one joint category of physical activity–BMI or another. For each joint category, life expectancy was defined as the age of 50% survival, and years of life gained were calculated as the difference in life expectancy from that of the reference group. Life expectancy models used age as the underlying time scale and were adjusted for gender, alcohol consumption (0, 0.1–14.9, 15.0–29.9, 30.0+ g/d), education (did not complete high school, completed high school, post-high-school training, some college, completed college), and marital status (married, divorced, widowed, unmarried).
(TIF)

Figure S9 **Multivariate hazard ratios of mortality in relation to joint categories of physical activity level and body mass index among healthy never smokers.** HRs are shown for participants aged 40 y or older with no history of smoking, heart disease, or cancer and with a BMI of at least 18.5 kg/m^2^ (*n* = 236,828; deaths = 16,074). The bars indicate the HRs for each joint category, and the vertical lines are the 95% CIs. The reference category is normal weight and 7.5+ MET-h/wk of physical activity (i.e., meeting US recommended physical activity levels). The multivariable HRs were calculated in models stratified by study that used age as the underlying time metric. Models were adjusted for gender, alcohol consumption (0, 0.1–14.9, 15.0–29.9, 30.0+ g/d), education (did not complete high school, completed high school, post-high-school training, some college, completed college), marital status (married, divorced, widowed, unmarried), and BMI (<18.5, 18.5–19.9, 20–22.4, 22.5–24.9, 25–27.4, 27.5–29.9, 30+ kg/m^2^).
(TIF)

Table S1 **Leisure time physical activity and life expectancies for participants aged 40+ y.**
(DOCX)

Table S2 **Leisure time physical activity and hazard ratio of mortality according to age at baseline.**
(DOCX)

Table S3 ***I*** **^2^ and** ***p*** **-value for heterogeneity for each physical activity level in random effects meta-analysis.** All studies together, and after excluding the Women's Health Study and the Women's Lifestyle and Health study.
(DOCX)

Table S4 **Leisure time physical activity and hazard ratio of mortality: impact of omitting each cohort from the analysis.**
(DOCX)

Table S5 **Leisure time physical activity and years of life gained according to cohort.**
(DOCX)

## Abbreviations

BMI: body mass index
CI: confidence interval
CPS II: Cancer Prevention Study II Nutrition Cohort
HR: hazard ratio
MET: metabolic equivalent
MET-h/wk: metabolic equivalent hours per week
NIH: National Institutes of Health
WHO: World Health Organization
