# Supplementary figures and images for: Leisure Time Physical Activity of Moderate to Vigorous Intensity and Mortality: A Large Pooled Cohort Analysis
Source: PLoS Med. 2012 Nov 6;9(11):e1001335. doi: 10.1371/journal.pmed.1001335 (PMC3491006; doi:10.1371/journal.pmed.1001335)

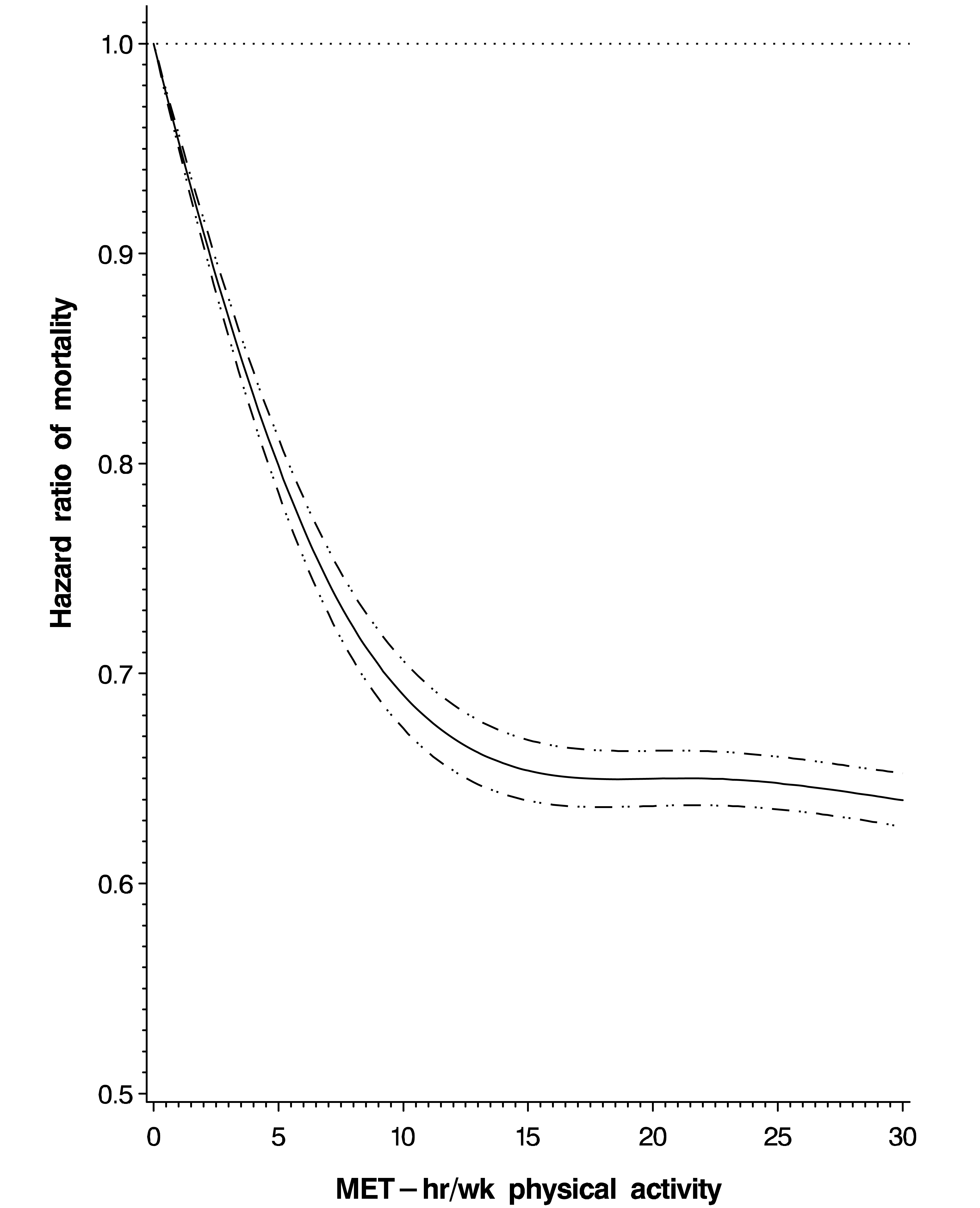

Supplement: Figure S1 — Leisure time physical activity and hazard ratios of mortality. In this figure, the line is a natural cubic spline [28] showing the shape of the dose–response curve for the association between physical activity and risk of mortality. HRs are indicated by the solid line, and 95% CIs by the dashed lines. The reference point is 0.0 MET-h/wk, with knots at 0, 7.5, 21.6, and 30.3 MET-h/wk. The graphic display is truncated at 30.0 MET-h/wk, which is the 95th percentile of the physical activity distribution. All models are adjusted for gender, alcohol consumption (0, 0.1–14.9, 15.0–29.9, 30.0+ g/d), education (did not complete high school, completed high school, post-high-school training, some college, completed college), marital status (married, divorced, widowed, unmarried), history of heart disease, history of cancer, BMI (<18.5, 1.8–19.9, 20–22.4, 22.5–24.9, 25–27.4, 27.5–29.9, 30+ kg/m2), and smoking status (never, former, current). (TIF) [file pmed.1001335.s001.tif]

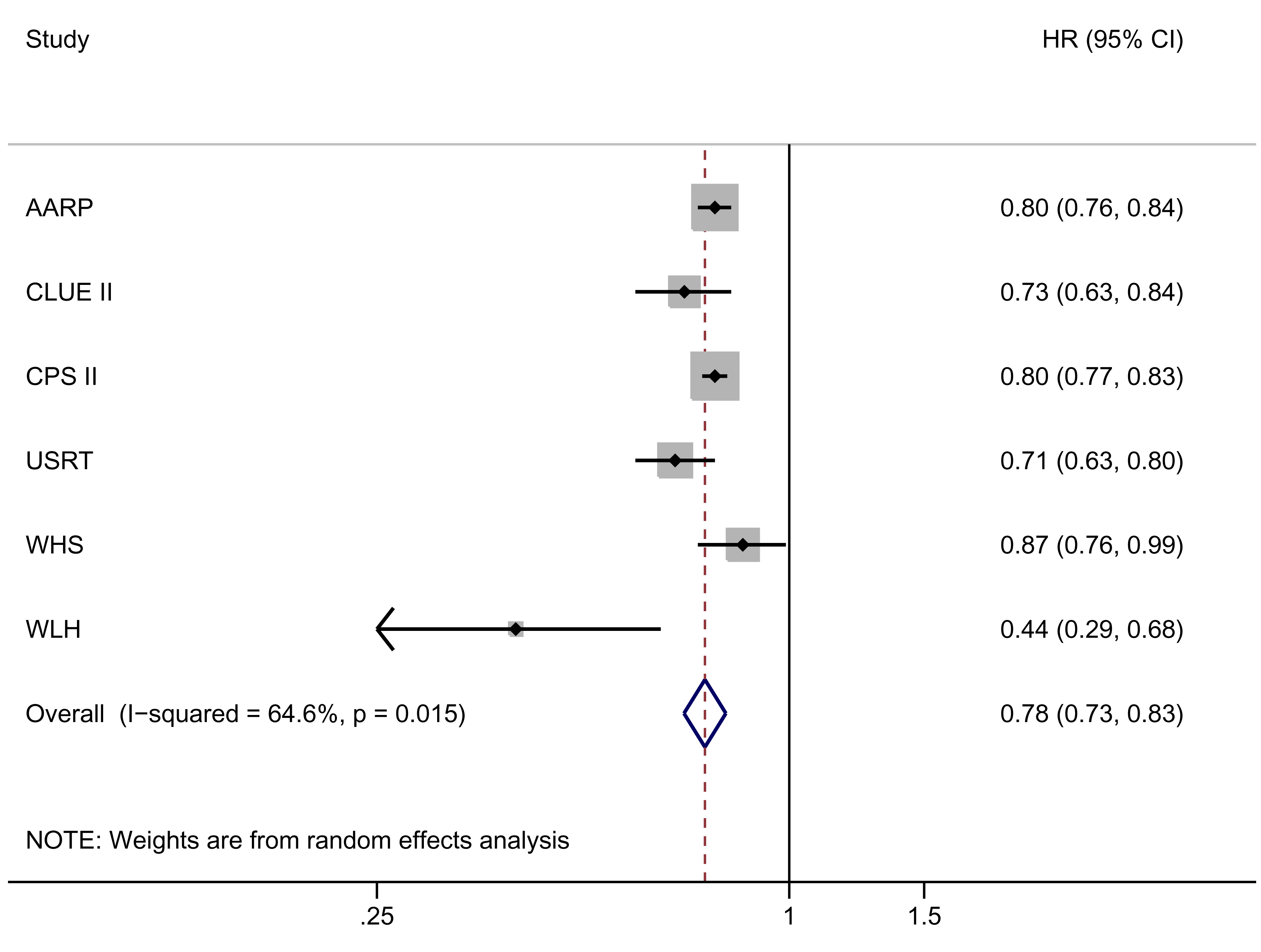

Supplement: Figure S2 — Forest plot of association between leisure time physical activity and mortality: 0.1–3.74 MET-h/wk versus 0.0 MET-h/wk. HRs are indicated by the box, and the size of the box is inversely proportional to the variance of the log HR estimate in each cohort. The lines show the 95% CIs. Models are adjusted for gender, alcohol consumption (0, 0.1–14.9, 15.0–29.9, 30.0+ g/d), education (did not complete high school, completed high school, post-high-school training, some college, completed college), marital status (married, divorced, widowed, unmarried), history of heart disease, history of cancer, BMI (<18.5, 1.8–19.9, 20–22.4, 22.5–24.9, 25–27.4, 27.5–29.9, 30+ kg/m2), and smoking status (never, former, current). (TIF) [file pmed.1001335.s002.tif]

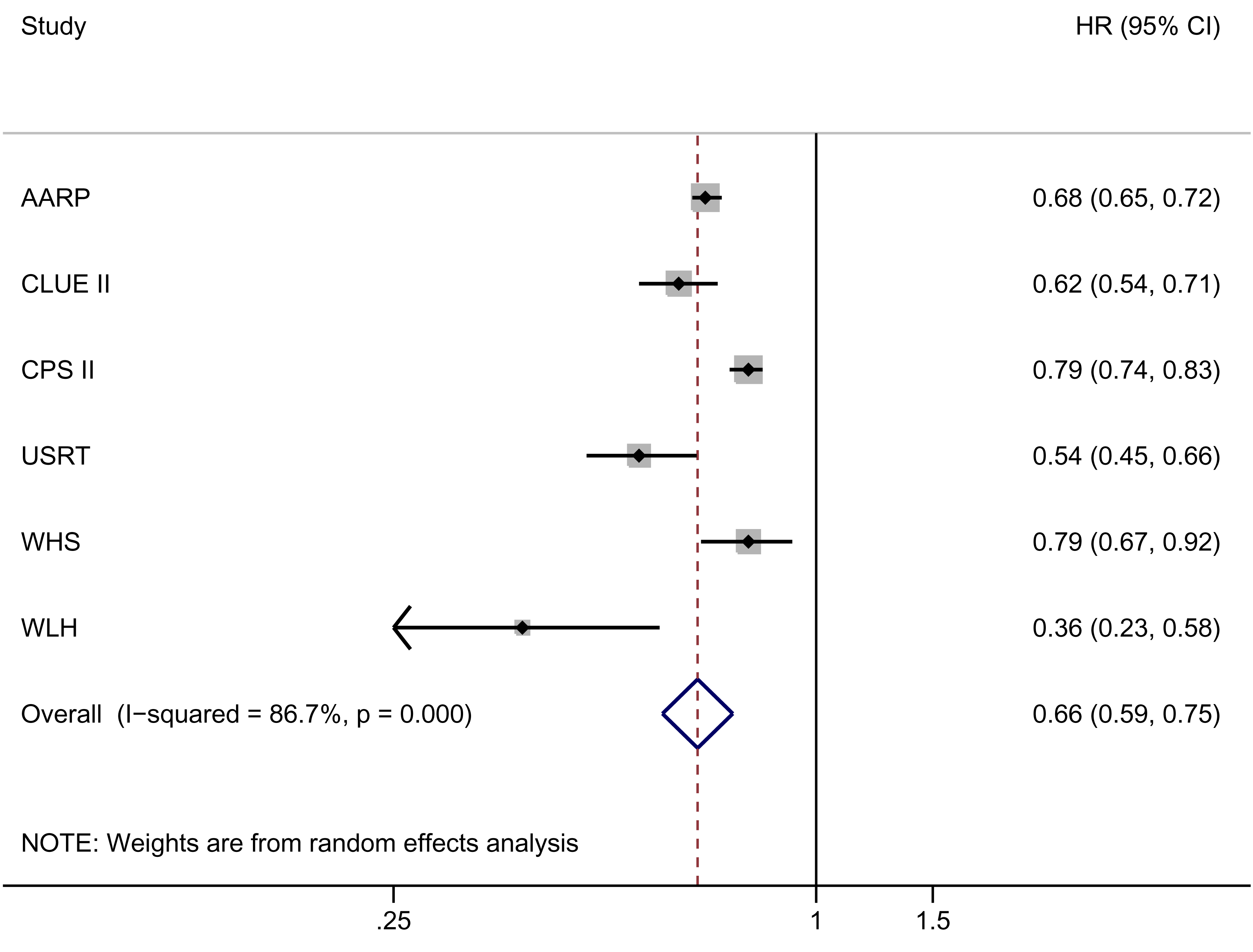

Supplement: Figure S3 — Forest plot of association between leisure time physical activity and mortality: 3.75–7.4 MET-h/wk versus 0.0 MET-h/wk. HRs are indicated by the box, and the size of the box is inversely proportional to the variance of the log HR estimate in each cohort. The lines show the 95% CIs. Models are adjusted for gender, alcohol consumption (0, 0.1–14.9, 15.0–29.9, 30.0+ g/d), education (did not complete high school, completed high school, post-high-school training, some college, completed college), marital status (married, divorced, widowed, unmarried), history of heart disease, history of cancer, BMI (<18.5, 1.8–19.9, 20–22.4, 22.5–24.9, 25–27.4, 27.5–29.9, 30+ kg/m2), and smoking status (never, former, current). (TIF) [file pmed.1001335.s003.tif]

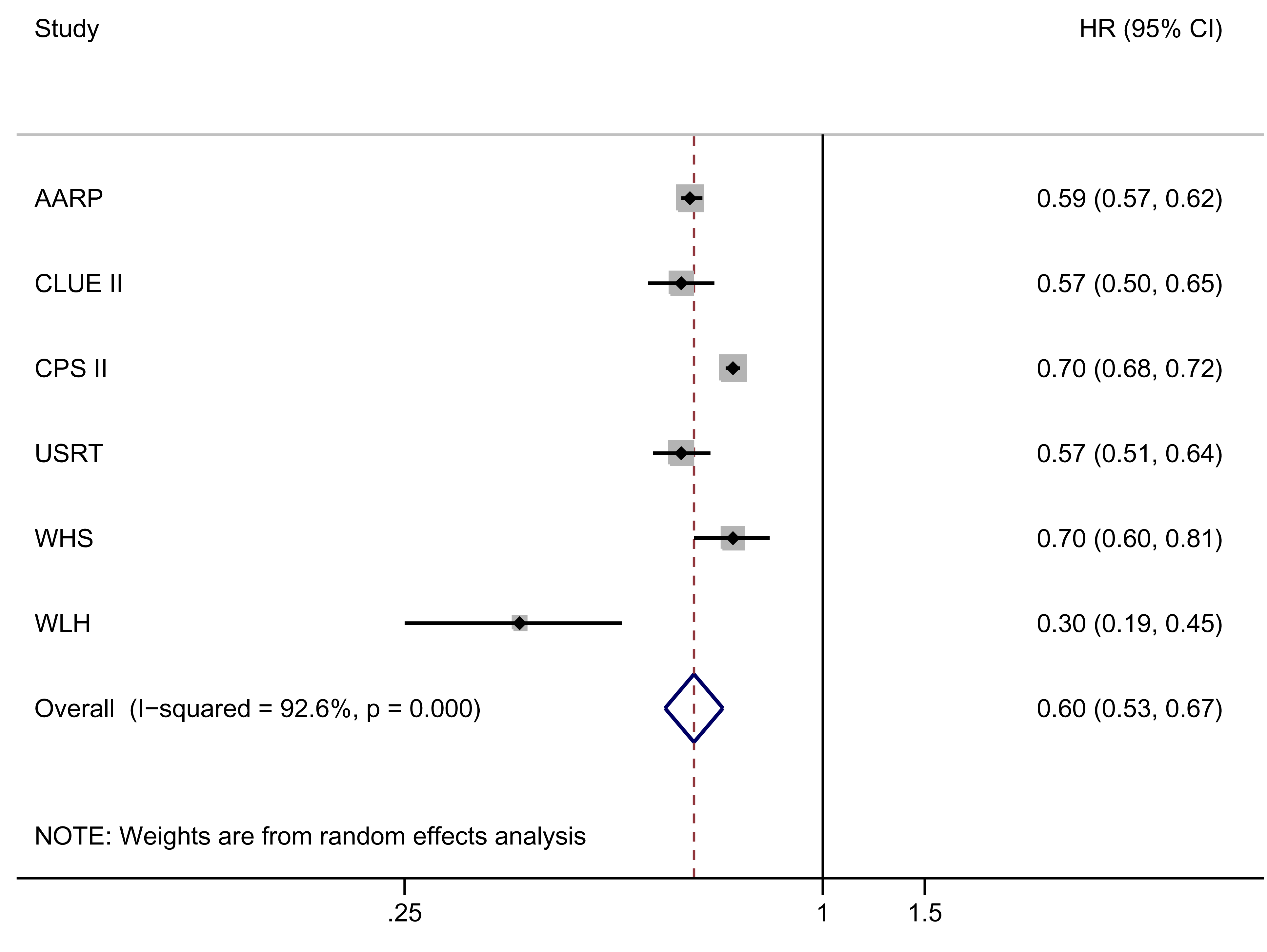

Supplement: Figure S4 — Forest plot of association between leisure time physical activity and mortality: 7.5–14.9 MET-h/wk versus 0.0 MET-h/wk. HRs are indicated by the box, and the size of the box is inversely proportional to the variance of the log HR estimate in each cohort. The lines show the 95% CIs. Models are adjusted for gender, alcohol consumption (0, 0.1–14.9, 15.0–29.9, 30.0+ g/d), education (did not complete high school, completed high school, post-high-school training, some college, completed college), marital status (married, divorced, widowed, unmarried), history of heart disease, history of cancer, BMI (<18.5, 1.8–19.9, 20–22.4, 22.5–24.9, 25–27.4, 27.5–29.9, 30+ kg/m2), and smoking status (never, former, current). (TIF) [file pmed.1001335.s004.tif]

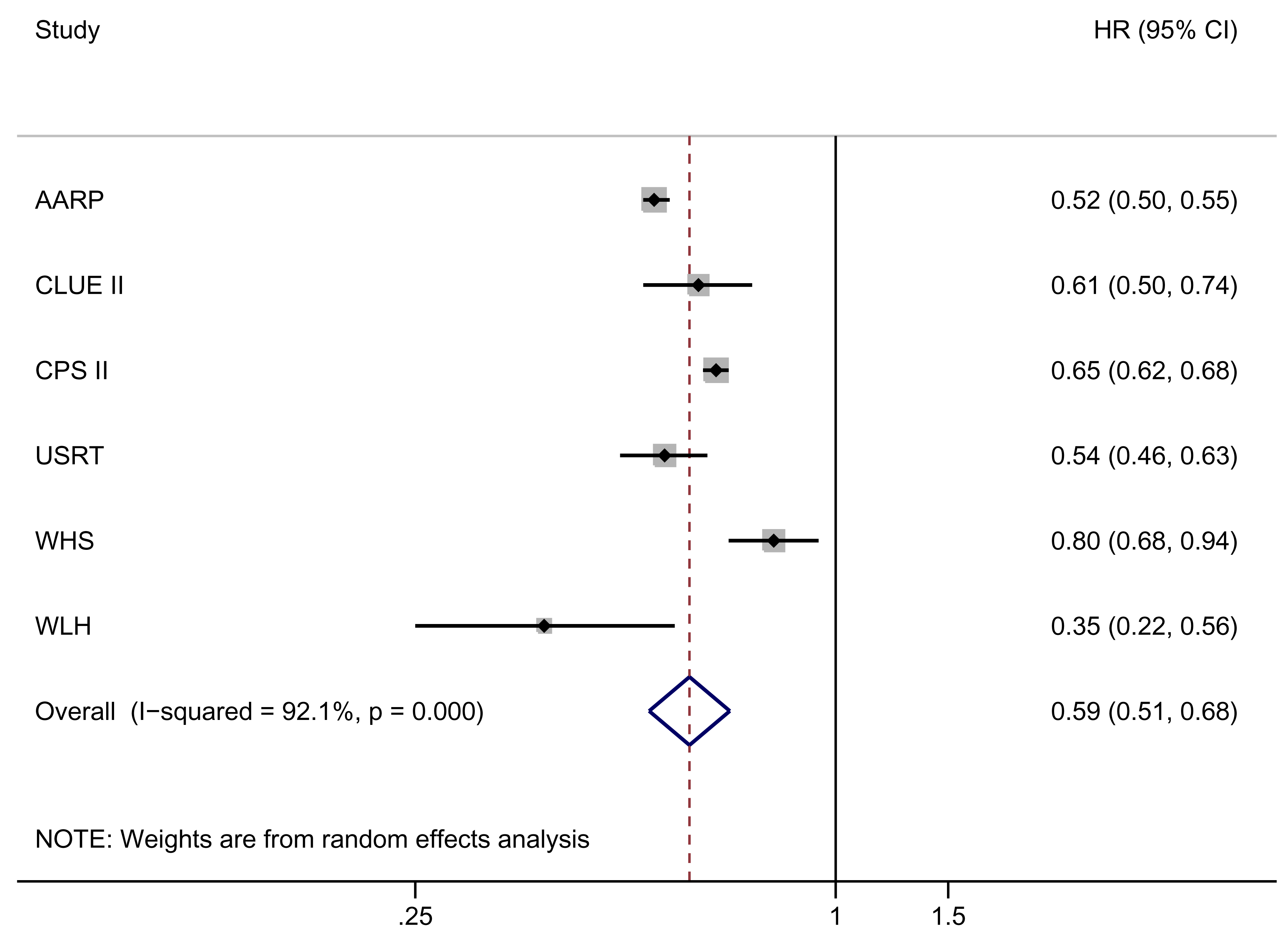

Supplement: Figure S5 — Forest plot of association between leisure time physical activity and mortality: 15.0–22.4 MET-h/wk versus 0.0 MET-h/wk. HRs are indicated by the box, and the size of the box is inversely proportional to the variance of the log HR estimate in each cohort. The lines show the 95% CIs. Models are adjusted for gender, alcohol consumption (0, 0.1–14.9, 15.0–29.9, 30.0+g/d), education (did not complete high school, completed high school, post-high-school training, some college, completed college), marital status (married, divorced, widowed, unmarried), history of heart disease, history of cancer, BMI (<18.5, 1.8–19.9, 20–22.4, 22.5–24.9, 25–27.4, 27.5–29.9, 30+kg/m2), and smoking status (never, former, current). (TIF) [file pmed.1001335.s005.tif]

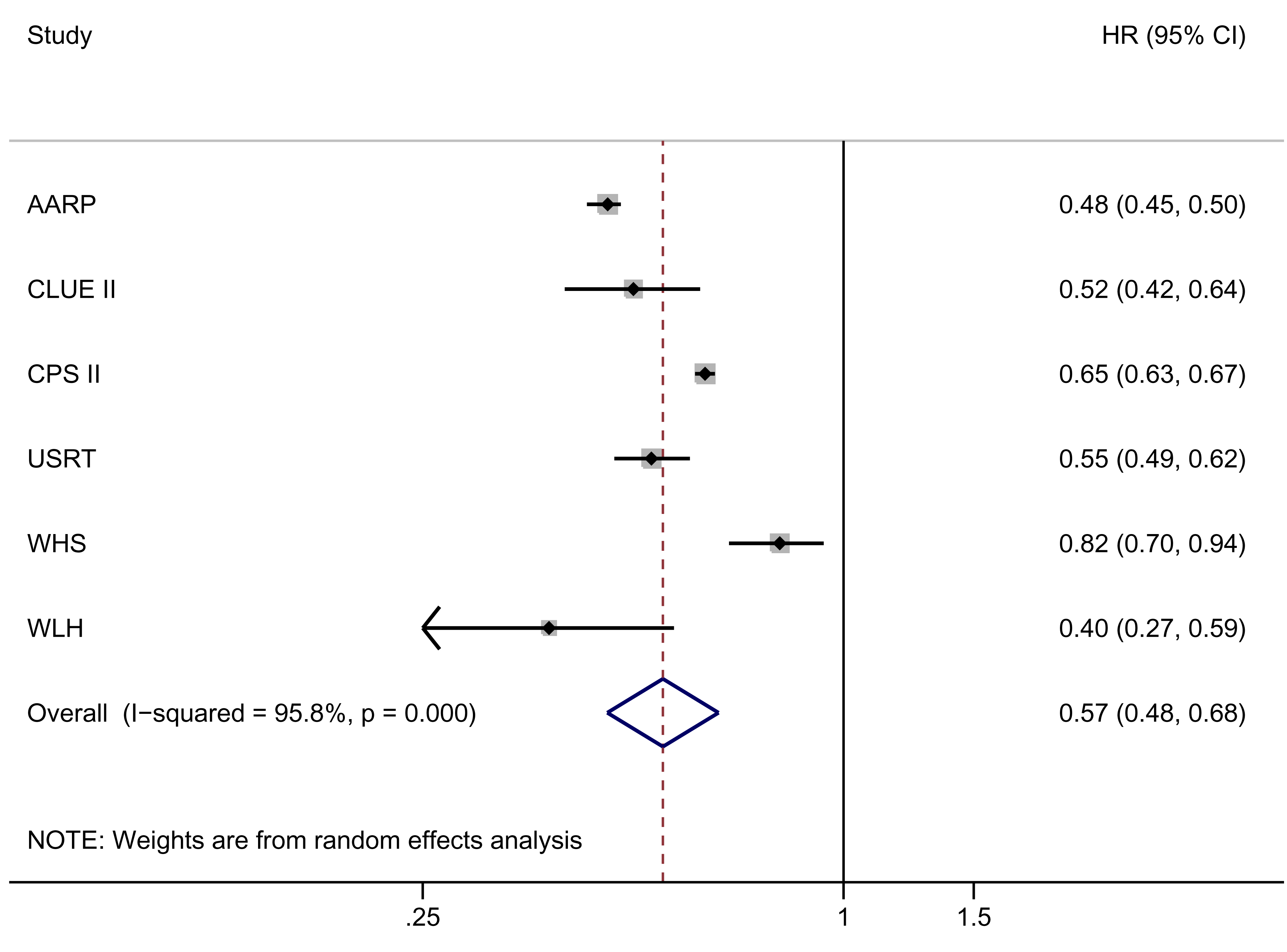

Supplement: Figure S6 — Forest plot of association between leisure time physical activity and mortality: 22.5+ MET-h/wk versus 0.0 MET-h/wk. HRs are indicated by the box, and the size of the box is inversely proportional to the variance of the log HR estimate in each cohort. The lines show the 95% CIs. Models are adjusted for gender, alcohol consumption (0, 0.1–14.9, 15.0–29.9, 30.0+g/d), education (did not complete high school, completed high school, post-high-school training, some college, completed college), marital status (married, divorced, widowed, unmarried), history of heart disease, history of cancer, BMI (<18.5, 1.8–19.9, 20–22.4, 22.5–24.9, 25–27.4, 27.5–29.9, 30+kg/m2), and smoking status (never, former, current). (TIF) [file pmed.1001335.s006.tif]

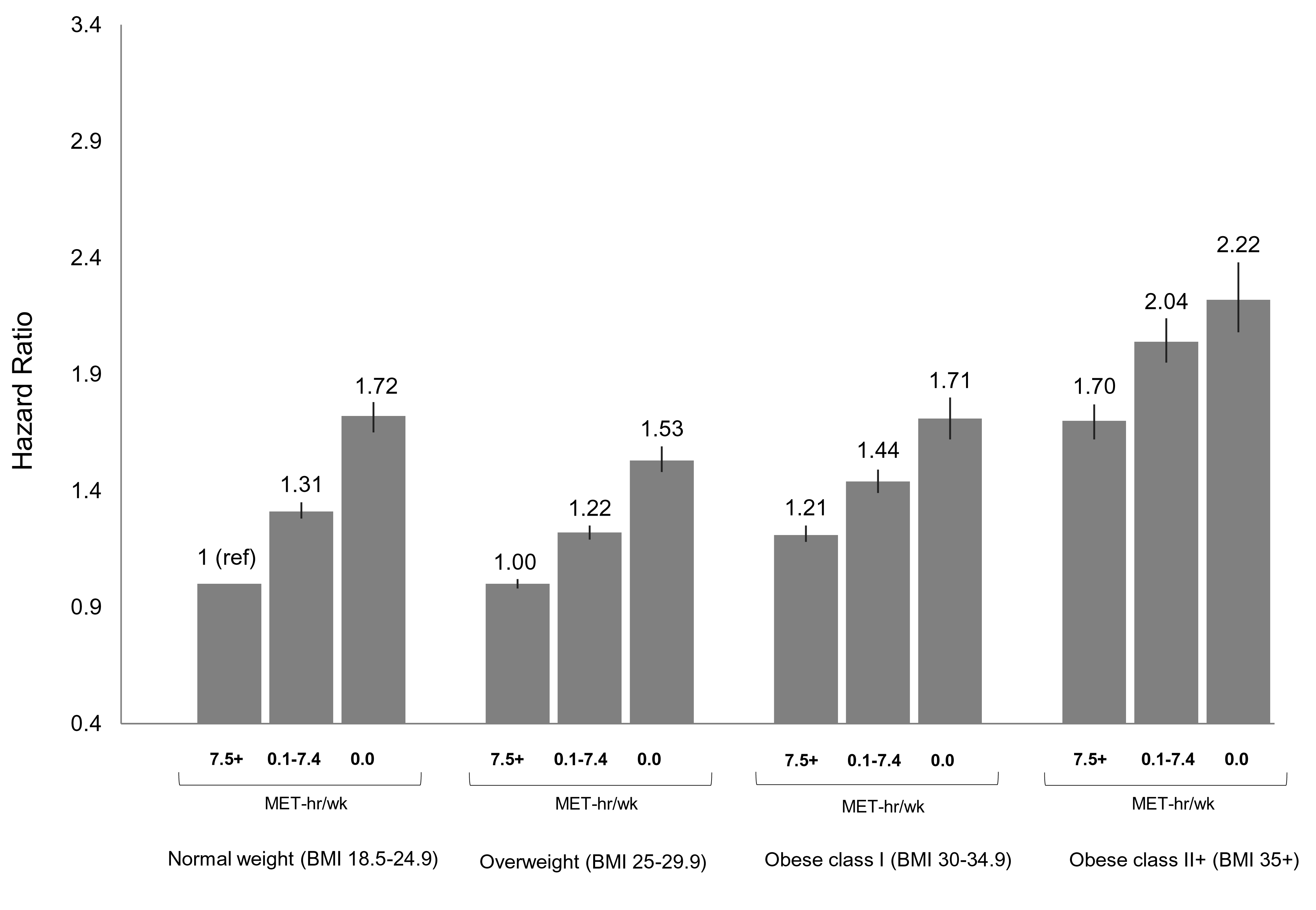

Supplement: Figure S7 — Hazard ratios of mortality in relation to joint categories of physical activity level and body mass index. HRs are shown for participants aged 40 y or older who had a BMI of at least 18.5 kg/m2 (n = 632,091; deaths = 80,767). The bars indicate the HRs for each joint category, and the vertical lines are the 95% CIs. The reference category is normal weight and 7.5+ MET-h/wk of physical activity (i.e., meeting US recommended physical activity levels). The multivariable HRs were calculated in models stratified by study that used age as the underlying time metric. Models were adjusted for gender, alcohol consumption (0, 0.1–14.9, 15.0–29.9, 30.0+ g/d), education (did not complete high school, completed high school, post-high-school training, some college, completed college), marital status (married, divorced, widowed, unmarried), history of heart disease, history of cancer, BMI (<18.5, 18.5–19.9, 20–22.4, 22.5–24.9, 25–27.4, 27.5–29.9, 30+ kg/m2), and smoking status (never, former, current). (TIF) [file pmed.1001335.s007.tif]

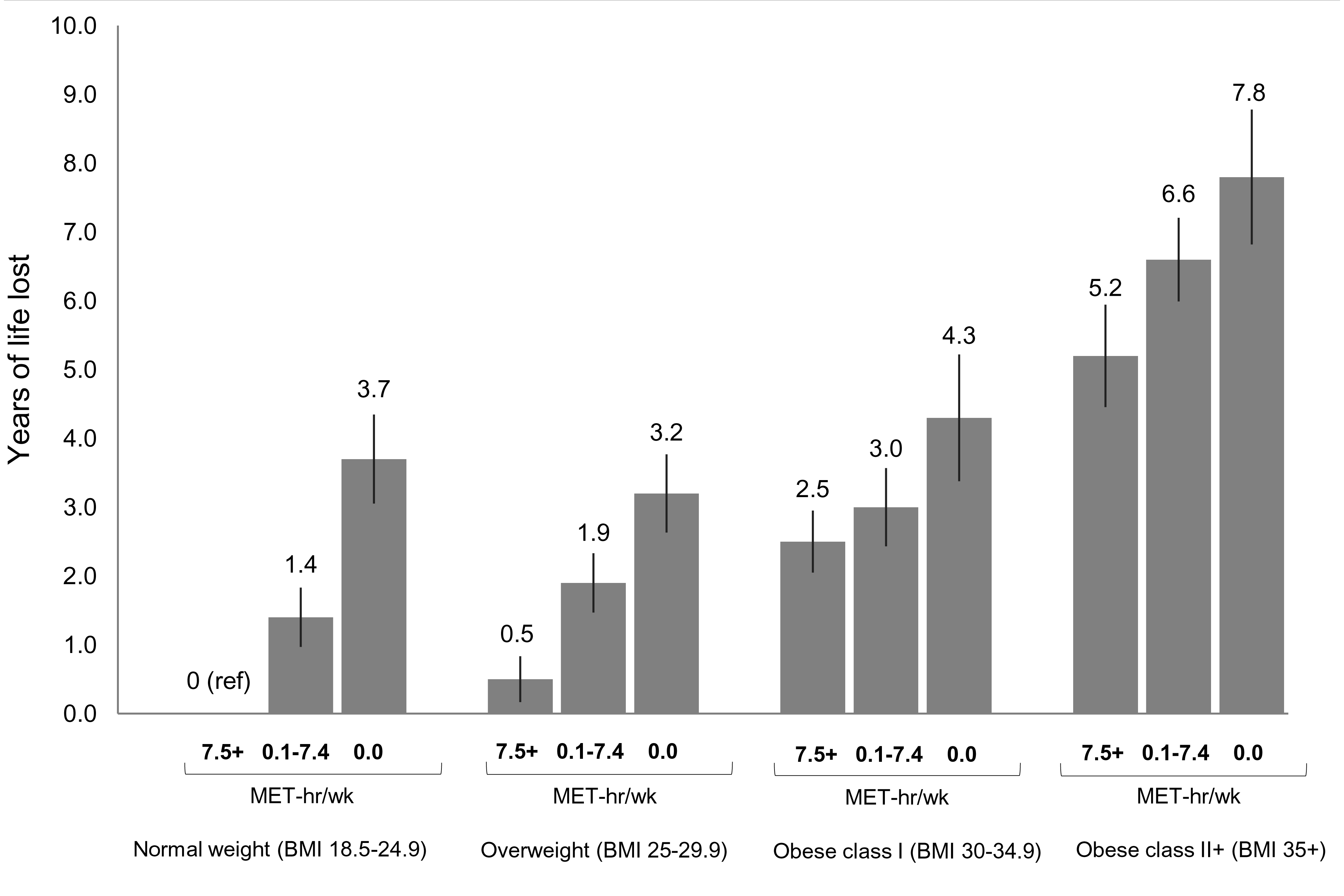

Supplement: Figure S8 — Years of life expectancy lost after age 40 in relation to joint categories of physical activity level and body mass index among healthy never smokers. Years of life expectancy gained/lost according to level of physical activity and BMI are shown for participants aged 40 y or older with no history of smoking, heart disease, or cancer and with a BMI of at least 18.5 kg/m2 (n = 236,828; deaths = 16,074). The bars indicate the number of years of life lost for each category, and the vertical lines are the 95% CIs. The reference category is normal weight and 7.5+ MET-h/wk of physical activity (i.e., meeting US recommended physical activity levels). Years of life expectancy lost after age 40 were derived using direct adjusted survival curves [31],[32] for participants who were 40+ y of age at baseline. By applying the hazard coefficient for each joint category to the entire study population, survival is estimated as if assigning all participants alternately to one joint category of physical activity–BMI or another. For each joint category, life expectancy was defined as the age of 50% survival, and years of life gained were calculated as the difference in life expectancy from that of the reference group. Life expectancy models used age as the underlying time scale and were adjusted for gender, alcohol consumption (0, 0.1–14.9, 15.0–29.9, 30.0+ g/d), education (did not complete high school, completed high school, post-high-school training, some college, completed college), and marital status (married, divorced, widowed, unmarried). (TIF) [file pmed.1001335.s008.tif]

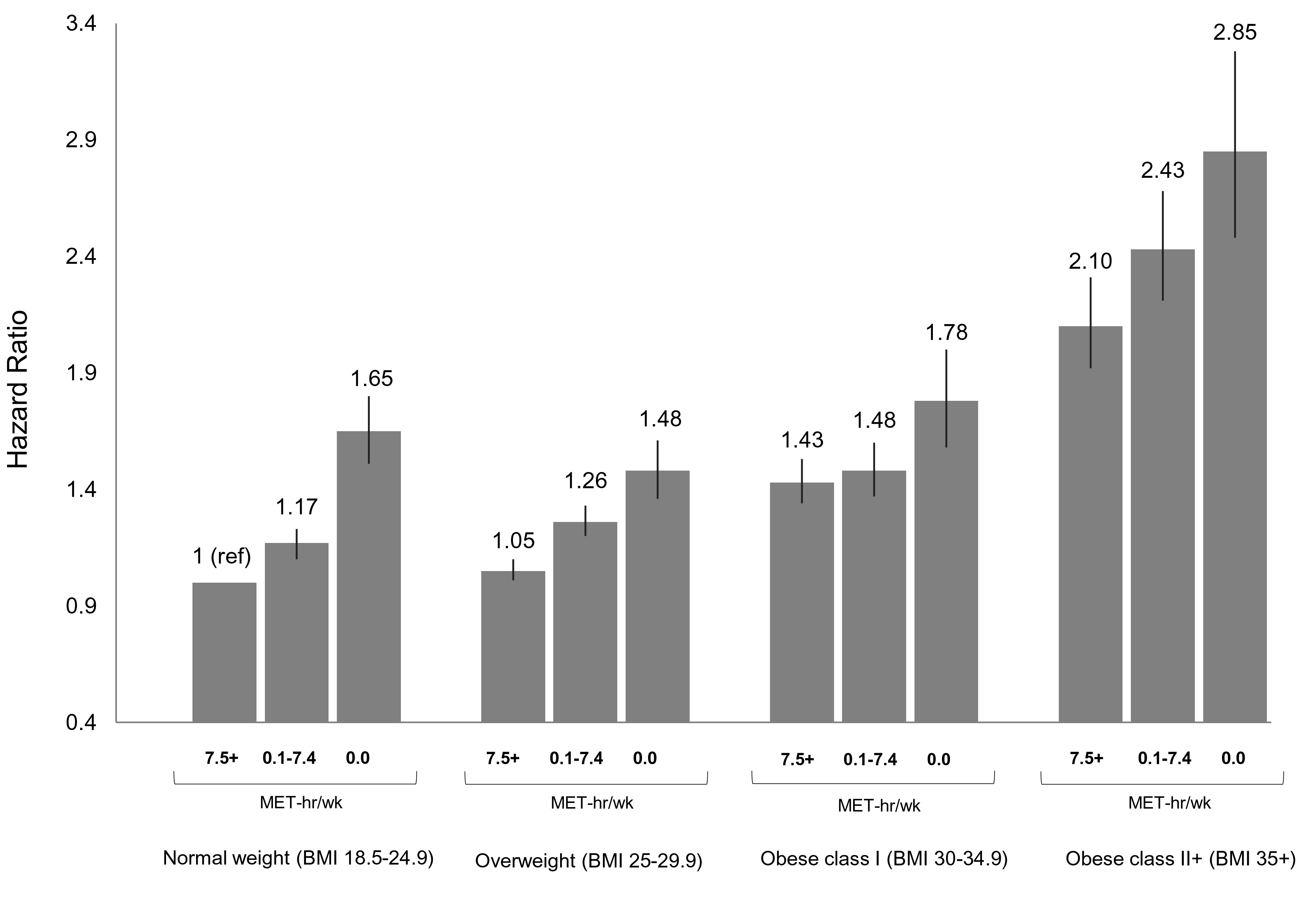

Supplement: Figure S9 — Multivariate hazard ratios of mortality in relation to joint categories of physical activity level and body mass index among healthy never smokers. HRs are shown for participants aged 40 y or older with no history of smoking, heart disease, or cancer and with a BMI of at least 18.5 kg/m2 (n = 236,828; deaths = 16,074). The bars indicate the HRs for each joint category, and the vertical lines are the 95% CIs. The reference category is normal weight and 7.5+ MET-h/wk of physical activity (i.e., meeting US recommended physical activity levels). The multivariable HRs were calculated in models stratified by study that used age as the underlying time metric. Models were adjusted for gender, alcohol consumption (0, 0.1–14.9, 15.0–29.9, 30.0+ g/d), education (did not complete high school, completed high school, post-high-school training, some college, completed college), marital status (married, divorced, widowed, unmarried), and BMI (<18.5, 18.5–19.9, 20–22.4, 22.5–24.9, 25–27.4, 27.5–29.9, 30+ kg/m2). (TIF) [file pmed.1001335.s009.tif]
