# Supplementary material for: Leisure Time Physical Activity of Moderate to Vigorous Intensity and Mortality: A Large Pooled Cohort Analysis
Source: PLoS Med. 2012 Nov 6;9(11):e1001335. doi: 10.1371/journal.pmed.1001335 (PMC3491006; doi:10.1371/journal.pmed.1001335)
Supplement: Table S1 — Leisure time physical activity and life expectancies for participants aged 40+ y. (DOCX) [file pmed.1001335.s010.docx]

**Table S1. Leisure time physical activity and life expectancies* for participants aged 40+ years**

|  | Physical activity level (MET-hr/wk) | | | | | |
| --- | --- | --- | --- | --- | --- | --- |
| Group† | 0 | 0.1-3.74 | 3.75-7.4 | 7.5-14.9 | 15.0-22.4 | 22.5+ |
|  |  |  |  |  |  |  |
| All participants | 84.6 (84.4, 84.8) | 86.4 (86.2, 86.6) | 87.0 (86.8, 87.3) | 88.0 (87.8, 88.1) | 88.8 (88.6, 89.1) | 89.1 (86.9, 89.3) |
| Men | 82.0 (81.7, 82.3) | 83.6 (83.4, 83.8) | 84.3 (84.0, 84.6) | 85.3 (85.1, 85.5) | 86.5 (86.3, 86.7) | 86.7 (86.4, 86.9) |
| Women | 86.4 (86.1, 86.8) | 88.5 (88.1, 88.9) | 89.2 (88.7, 89.6) | 90.0 (89.5, 90.5) | 90.5 (89.9, 91.1) | 91.0 (90.5, 91.4) |
| White | 84.6 (84.3, 84.8) | 86.4 (86.2, 86.6) | 87.0 (86.7, 87.3) | 87.9 (87.7, 88.1) | 88.9 (88.6, 89.1) | 89.1 (88.9, 89.3) |
| Black | 84.4 (82.1, 86.7) | 87.0 (85.6, 88.5) | 88.1 (86.4, 89.7) | 89.7 (87.9, 91.5) | 88.0 (86.4, 89.5) | 90.8 (88.6, 92.9) |
| High school/dropout | 83.4 (83.1, 83.7) | 85.1 (84.8, 85.4) | 85.9 (85.4, 86.5) | 86.8 (86.5, 87.2) | 87.4 (87.0, 87.7) | 87.7 (87.4, 88.1) |
| Some college | 84.3 (83.9, 84.7) | 86.5 (86.0, 86.9) | 87.1 (86.7, 87.5) | 88.0 (87.6, 88.4) | 89.0 (88.6, 89.4) | 89.4 (89.0, 89.7) |
| College graduate | 85.2 (84.8, 85.7) | 87.1 (86.8, 87.5) | 87.3 (86.9, 87.8) | 88.5 (88.1, 88.9) | 89.6 (89.2, 90.0) | 89.7 (89.3, 90.1) |
| Never smoker | 87.9 (87.4, 88.4) | 89.5 (89.2, 89.8) | 90.1 (89.5, 90.7) | 90.9 (90.6, 91.2) | 91.1 (90.7, 91.5) | 91.3 (90.9, 91.6) |
| Former smoker | 83.0 (82.7, 83.4) | 85.3 (85.0, 85.5) | 85.8 (85.4, 86.3) | 87.1 (86.9, 87.3) | 88.0 (87.8, 88.3) | 88.5 (88.3, 88.8) |
| Current smoker | 78.4 (77.9, 79.0) | 79.7 (79.3, 80.1) | 80.9 (80.3, 81.5) | 81.0 (80.3, 81.5) | 82.1 (81.6, 82.6) | 81.9 (81.4, 82.3) |
|  |  |  |  |  |  |  |

* Life expectancy models used age for the underlying time scale and were adjusted for gender, alcohol consumption (0, 0.1-14.9, 15.0-29.9 and 30.0+ g/day), education (did not complete high school, completed high school, post high-school training, some college, completed college), marital status (married, divorced, widowed, single, unmarried), history of heart-disease, history of cancer, body mass index (<18.5, 1.8-19.9, 20-22.4, 22.5-24.9, 25-27.4, 27.5-29.9, 30+), and smoking status (never, former, current). If a covariate is a stratification variable for a particular model, then it is excluded from multivariable adjustment.

† Within each demographic group, life expectancies are adjusted for covariate differences across physical activity levels through use of direct adjusted survival curves [31, 32]. However, covariates are not adjusted for across demographic groups, e.g. the proportion of men among never smokers may be different from that of current smokers.
