# Supplementary material for: Leisure Time Physical Activity of Moderate to Vigorous Intensity and Mortality: A Large Pooled Cohort Analysis
Source: PLoS Med. 2012 Nov 6;9(11):e1001335. doi: 10.1371/journal.pmed.1001335 (PMC3491006; doi:10.1371/journal.pmed.1001335)
Supplement: Table S2 — Leisure time physical activity and hazard ratio of mortality according to age at baseline. (DOCX) [file pmed.1001335.s011.docx]

**Table S2.  Leisure time physical activity and hazard ratio* (HR) of mortality according to age at baseline**

| **Variable** | Physical activity level (MET-hr/wk) | | | | | |
| --- | --- | --- | --- | --- | --- | --- |
|  |  |  |  |  |  |  |
|  | 0 | 0.1-3.74 | 3.75-7.4 | 7.5-14.9 | 15.0-22.4 | 22.5+ |
|  |  |  |  |  |  |  |
| **40-59 yr of age** |  |  |  |  |  |  |
| No. of deaths | 1,723 | 2,896 | 1,231 | 3,037 | 1,840 | 2,304 |
| Multivariate HR | 1.0 | 0.81 | 0.73 | 0.68 | 0.66 | 0.68 |
| 95% CI | ref | 0.76, 0.86 | 0.68, 0.79 | 0.64, 0.72 | 0.62, 0.71 | 0.64, 0.72 |
| **60-69 yr of age** |  |  |  |  |  |  |
| No. of deaths | 5,101 | 10,430 | 4,267 | 12,315 | 7,636 | 9,116 |
| Multivariate HR | 1.0 | 0.82 | 0.78 | 0.69 | 0.61 | 0.58 |
| 95% CI | ref | 0.80, 0.85 | 0.75, 0.82 | 0.67, 0.71 | 0.58, 0.63 | 0.56, 0.60 |
| **70-79 yr of age** |  |  |  |  |  |  |
| No. of deaths | 2,532 | 4,681 | 1,306 | 4,759 | 2,242 | 3,562 |
| Multivariate HR | 1.0 | 0.78 | 0.74 | 0.64 | 0.62 | 0.58 |
| 95% CI | ref | 0.75, 0.82 | 0.69, 0.80 | 0.61, 0.68 | 0.58, 0.65 | 0.55, 0.61 |
| **80+ yr of age** |  |  |  |  |  |  |
| No. of deaths | 380 | 338 | 154 | 298 | 76 | 148 |
| Multivariate HR | 1.0 | 0.74 | 0.66 | 0.64 | 0.52 | 0.56 |
| 95% CI | ref | 0.64, 0.87 | 0.54, 0.80 | 0.55, 0.75 | 0.40, 0.67 | 0.46, 0.69 |

* Hazard ratios were calculated in models stratified by study that used age as the underlying time-scale. Multivariable models were adjusted for gender, alcohol consumption (0, 0.1-14.9, 15.0-29.9 and 30.0+ g/day), education (did not complete high school, completed high school, post high-school training, some college, completed college), marital status (married, divorced, widowed, unmarried), history of heart-disease, history of cancer, body mass index (<18.5, 18.5-19.9, 20-22.4, 22.5-24.9, 25-27.4, 27.5-29.9, 30+), and smoking status (never, former, current).
