# Supplementary material for: Leisure Time Physical Activity of Moderate to Vigorous Intensity and Mortality: A Large Pooled Cohort Analysis
Source: PLoS Med. 2012 Nov 6;9(11):e1001335. doi: 10.1371/journal.pmed.1001335 (PMC3491006; doi:10.1371/journal.pmed.1001335)
Supplement: Table S3 — I 2 and p -value for heterogeneity for each physical activity level in random effects meta-analysis. All studies together, and after excluding the Women's Health Study and the Women's Lifestyle and Health study. (DOCX) [file pmed.1001335.s012.docx]

**Table S3. I^2^ and P-value for heterogeneity for each physical activity level in random effects meta-analysis: all studies together, and after excluding WLH and WHS.**

|  |  |  | Physical activity level (MET-hr/wk) | | | |  |  |
| --- | --- | --- | --- | --- | --- | --- | --- | --- |
|  |  | 0 | 0.1-3.74 | 3.75-7.4 | 7.5-14.9 | 15.0-22.4 | | 22.5+ |
| All studies | | - | 64.6% (0.02) | 86.7% (<0.01) | 92.6% (<0.01) | 92.1% (<0.01) | | 95.8% (<0.01) |
| All except WLH | | - | 40.7% (0.15) | 86.3% (<0.01) | 92.8% (<0.01) | 93.2% (<0.01) | | 96.5% (<0.01) |
| All except WLH and WHS | | - | 38.5% (0.18) | 89.1% (<0.01) | 94.5% (<0.01) | 93.3% (<0.01) | | 97.0% (<0.01) |
