# Supplementary material for: Leisure Time Physical Activity of Moderate to Vigorous Intensity and Mortality: A Large Pooled Cohort Analysis
Source: PLoS Med. 2012 Nov 6;9(11):e1001335. doi: 10.1371/journal.pmed.1001335 (PMC3491006; doi:10.1371/journal.pmed.1001335)
Supplement: Table S4 — Leisure time physical activity and hazard ratio of mortality: impact of omitting each cohort from the analysis. (DOCX) [file pmed.1001335.s013.docx]

**Table S4. Leisure time physical activity and hazard ratio* of mortality: impact of omitting each cohort from the analysis**

|  |  |  |  | Physical activity level (MET-hr/wk) | | |  |  |
| --- | --- | --- | --- | --- | --- | --- | --- | --- |
| Omitted study† |  |  | 0 | 0.1-3.74 | 3.75-7.4 | 7.5-14.9 | 15.0-22.4 | 22.5+ |
|  |  |  |  |  |  |  |  |  |
| AARP |  |  | 1.0 (ref) | 0.79 (0.77, 0.82) | 0.75 (0.72, 0.79) | 0.69 (0.67, 0.71) | 0.66 (0.63, 0.69) | 0.65 (0.63, 0.67) |
| CLUE II |  |  | 1.0 (ref) | 0.81 (0.79, 0.84) | 0.77 (0.74, 0.79) | 0.68 (0.66, 0.70) | 0.61 (0.60, 0.63) | 0.59 (0.58, 0.61) |
| CPS II |  |  | 1.0 (ref) | 0.81 (0.78, 0.84) | 0.70 (0.67, 0.73) | 0.62 (0.59, 0.64) | 0.55 (0.53, 0.58) | 0.51 (0.49, 0.53) |
| USRT |  |  | 1.0 (ref) | 0.82 (0.80, 0.84) | 0.77 (0.74, 0.79) | 0.68 (0.66, 0.70) | 0.62 (0.60, 0.63) | 0.59 (0.58, 0.61) |
| WHS |  |  | 1.0 (ref) | 0.81 (0.79, 0.83) | 0.76 (0.73, 0.78) | 0.67 (0.66, 0.69) | 0.61 (0.59, 0.62) | 0.58 (0.57, 0.60) |
| WLH |  |  | 1.0 (ref) | 0.81 (0.79, 0.83) | 0.76 (0.74, 0.79) | 0.68 (0.66, 0.69) | 0.61 (0.60, 0.63) | 0.59 (0.58, 0.61) |
|  |  |  |  |  |  |  |  |  |
| All studies | |  | 1.0 (ref) | 0.81 (0.79, 0.83) | 0.76 (0.74, 0.78) | 0.68 (0.66, 0.69) | 0.61 (0.59, 0.63) | 0.59 (0.57, 0.61) |

* Hazard ratios were calculated in models stratified by study that used age as the underlying time-scale. Multivariable models were adjusted for gender, alcohol consumption (0, 0.1-14.9, 15.0-29.9 and 30.0+ g/day), education (did not complete high school, completed high school, post high-school training, some college, completed college), marital status (married, divorced, widowed, unmarried), history of heart-disease, history of cancer, body mass index (<18.5, 18.5-19.9, 20-22.4, 22.5-24.9, 25-27.4, 27.5-29.9, 30+), and smoking status (never, former, current).

† Abbreviations. AARP: NIH-AARP Diet and Health Study, CLUE II: CLUE II, CPS II: Cancer Prevention Study II, USRT: U.S. Radiologic Technologists Cohort, WHS: Women's Health Study, WLH: Women's Lifestyle and Health Study
