# Supplementary material for: Leisure Time Physical Activity of Moderate to Vigorous Intensity and Mortality: A Large Pooled Cohort Analysis
Source: PLoS Med. 2012 Nov 6;9(11):e1001335. doi: 10.1371/journal.pmed.1001335 (PMC3491006; doi:10.1371/journal.pmed.1001335)
Supplement: Table S5 — Leisure time physical activity and years of life gained according to cohort. (DOCX) [file pmed.1001335.s014.docx]

**Table S5. Leisure time physical activity and years of life gained* according to cohort**

|  |  |  |  | Physical activity level (MET-hr/wk) | | |  |  |
| --- | --- | --- | --- | --- | --- | --- | --- | --- |
| Study‡ |  |  | 0 | 0.1-3.74 | 3.75-7.4 | 7.5-14.9 | 15.0-22.4 | 22.5+ |
|  |  |  |  |  |  |  |  |  |
| AARP |  |  | N/A† | | | | | |
| CLUE II |  |  | 0 (ref) | 2.6 (1.8, 3.4) | 3.4 (2.6, 4.2) | 3.6 (3.0, 4.3) | 3.3 (2.1, 4.5) | 3.9 (2.6, 5.2) |
| CPS II |  |  | 0 (ref) | 2.2 (2.0, 2.4) | 2.3 (1.9, 2.8) | 3.2 (3.0, 3.4) | 3.8 (3.4, 4.1) | 3.5 (3.2, 3.7) |
| USRT |  |  | 0 (ref) | 3.4 (2.2, 4.7) | 6.2 (4.3, 8.1) | 6.0 (4.7, 7.4) | 6.5 (4.5, 8.5) | 6.0 (4.4, 7.5) |
| WHS |  |  | 0 (ref) | 0.9 (-0.3, 2.0) | 1.3 (-0.5, 3.0) | 2.1 (0.3, 3.9) | 1.2 (-0.6, 3.0) | 1.1 (-0.5, 2.6) |
| WLH |  |  | N/A† | | | | | |
|  |  |  |  |  |  |  |  |  |

* Life expectancy models were adjusted for age at study enrollment, gender, alcohol consumption (0, 0.1-14.9, 15.0-29.9 and 30.0+ g/day), education (did not complete high school, completed high school, post high-school training, some college, completed college), marital status (married, divorced, widowed, single, unmarried), history of heart-disease, history of cancer, body mass index (<18.5, 1.8-19.9, 20-22.4, 22.5-24.9, 25-27.4, 27.5-29.9, 30+), and smoking status (never, former, current).

† N/A: not applicable. The years of life gained could not be directly estimated for the AARP and WLH cohorts due to sparse data at older ages, although these cohorts do contribute to the pooled analysis. Based on hazard ratio estimates, the years of life gained for the AARP and WLH cohorts may be somewhat larger than those for the average of the cohorts shown.

‡ Abbreviations. AARP: NIH-AARP Diet and Health Study, CLUE II: CLUE II, CPS II: Cancer Prevention Study II, USRT: U.S. Radiologic Technologists Cohort, WHS: Women's Health Study, WLH: Women's Lifestyle and Health Study
